# Oxide semiconductor based deep‐subthreshold operated read‐out electronics for all‐printed smart sensor patches

**DOI:** 10.1002/EXP.20230167

**Published:** 2024-05-15

**Authors:** Jyoti Ranjan Pradhan, Sushree Sangita Priyadarsini, Sanjana R. Nibgoor, Manvendra Singh, Subho Dasgupta

**Affiliations:** ^1^ Department of Materials Engineering Indian Institute of Science (IISc) Bangalore Karnataka India

**Keywords:** analog‐to‐digital converter, inkjet printing, printed oxide electronics, printed read‐out electronics, smart sensor patches, thin film transistors

## Abstract

The ability to fabricate an entire smart sensor patch with read‐out electronics using commercial printing techniques may have a wide range of potential applications. Although solution‐processed oxide thin film transistors (TFTs) are capable of providing high mobility electron transport, resulting in large ON‐state current and power output, there is hardly any literature report that uses the printed oxide TFTs at the sensor interfaces. Here, printed amorphous indium‐gallium‐zinc oxide (*a*‐IGZO)‐based deep‐subthreshold operated TFTs that comprise signal amplifiers and analog‐to‐digital converters (ADCs) that can successfully digitalize the analog sensor signals up to a frequency range of 1 kHz are reported. In addition, exploiting the high current oxide TFTs, a current drive circuit placed after the ADC unit has been found useful in producing easy‐to‐detect visual recognition of the sensor signal at a predefined threshold crossover. Notably, the entire smart sensor patch is demonstrated to operate at a low supply voltage of ≤2 V, thereby ensuring that it can be an on‐chip energy source compatible and standalone detection unit.

## Graphical abstract

The readout circuit based on inkjet‐printed deep‐subthreshold operated thin film transistors (TFTs) based on amorphous indium‐gallium‐zinc oxide (*a*‐IGZO) successfully digitalizes analog sensor data and provides easy‐to‐detect visual identification of the sensor signal at a preset threshold crossover using high‐current oxide TFTs. Notably, the complete smart sensor patch is shown to operate at a low voltage of ≤2 V, assuring that the on‐chip power source is compatible.

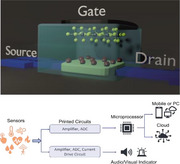

## 1 INTRODUCTION

Emerging electronics beyond Si‐MOSFETs, which include solution‐processed/printed and flexible electronics as well, are showing an ever‐increasing demand for getting included in daily‐life applications.^[^ ^1^, ^2^, ^3^, ^4^, ^5^, ^6^, ^7^ ^]^ In this regard, Internet of Things (IoT) or smart sensor systems, for example, bio‐sensors/artificial muscles,^[^ ^8^ ^]^ blood coagulation testing systems,^[^ ^9^ ^]^ virus diagnosis,^[^ ^10^ ^]^ photo detectors,^[^ ^11^, ^12^, ^13^ ^]^ wearable sensors for healthcare,^[^ ^14^, ^15^, ^16^ ^]^ e‐skins^[^ ^17^, ^18^ ^]^ etc., have emerged, which are again often powered by solution‐processed solar cells^[^ ^19^, ^20^ ^]^ or triboelectric nanogenerators (TENG).^[^ ^21^, ^22^ ^]^ Here, the motivation for solution processing/printing is that a high volume of device production would be possible, and a large range of products would be easy to encompass.^[^ ^23^, ^24^, ^25^, ^26^ ^]^ However, when focussing on fully‐printed circuits or sensor patches, the previous literature reports are quite limited.^[^ ^27^, ^28^, ^29^, ^30^ ^]^ It may be related to the fact that the complexity and reliability of such fully‐printed smart tag demands are not easy to realize with alternative device fabrication techniques, such as printing. For example, a smart sensor tag would require reliable read‐out electronics and wireless communication to transfer the sensor signal which is certainly non‐trivial to achieve using solution‐processed TFTs. The key component of an analog front‐end, that is, the read‐out electronics, is the analog‐to‐digital converter (ADC), which is already rare to have fabricated using printing techniques. In fact, the previous reports on amorphous oxide‐based ADCs were all realized using vacuum deposition techniques, mostly sputtering.^[^ ^31^, ^32^, ^33^, ^34^ ^]^ Moreover, in these cases, the ADC designs are quite complex, which may not be easy to replicate when TFTs and the other components are printed. On the other hand, these circuits are designed in such a way that the supply voltage requirement is greater than 10 V, which is not compatible with the envisioned power source on‐chip and fully printed smart sensor tags. On the other hand, examples of solution‐processed ADCs can be found in the literature that are based on organic semiconductors; however, again, they require an operation voltage of 15–20 V.^[^ ^35^, ^36^ ^]^

In an attempt to simplify the readout electronics, one may consider a much simpler ADC design and an easy‐to‐detect, on‐chip, audio‐visual recognition of the sensor signal, as opposed to a complex wireless communication of the digitalized data. However, such an audio‐visual demonstration of the sensor signal or the ADC output, be it illumination based, a chemical reaction‐induced colour change of a material, or a small motorized mechanical movement, would always require a supply of large currents. On the one hand, this necessitates a current drive circuit to be placed in series with the ADC unit; on the other hand, the large current requirement ensures that the oxide TFTs become a natural choice ahead of their organic counterparts.^[^ ^37^, ^38^, ^39^, ^40^, ^41^, ^42^, ^43^ ^]^ However, within the oxide TFTs, there is also a serious performance mismatch between the high‐performing *n*‐type and the low‐mobility *p*‐type transistors.^[^ ^7^, ^44^, ^45^ ^]^ Hence, it has often been found that all *n*‐type unipolar logics perform way superiorly to all‐oxide CMOS electronics.^[^ ^46^, ^47^, ^48^, ^49^, ^50^, ^51^, ^52^, ^53^ ^]^ Nevertheless, it is also important to note that in the case of the proposed standalone smart sensor tag, the on‐chip energy source would rather be limited, and consequently, the static, as well as the dynamic power dissipation of the unipolar logics should not be too high. In this regard, it is known that Schottky contact TFTs can provide high gain with ultra‐low power consumption when operated at the deep‐subthreshold regime, that is, near the off‐state of the transistors.^[^ ^54^, ^55^, ^56^ ^]^ This strategy has been adopted to fabricate the high gain amplifiers and ADC units, where the DRIVE TFTs are always operated near the off‐state of the transistors. The steep transfer curve at the deep‐subthreshold regime also helps to attain a large signal gain of 140 and excellent noise immunity for the printed inverters, which are then used to realize common‐source and differential amplifiers that can amplify the sinusoidal signals with an amplification ratio of 120, and up to an operation frequency of 1 kHz. Therefore, in this study, we primarily demonstrate a sensor, a high‐signal gain amplifier, an extremely simple design 1‐bit ADC unit with only four printed transistors, and a current drive circuit that is fed with the ADC output and connected to a chemically‐induced colour change‐based visual recognition unit, all printed using a commercial inkjet printer. Here, the visual recognition unit, which is comprised of two printed silver electrodes and a hydrochloric acid based solid polymer electrolyte, turns the shiny silvery colour of the first electrode to blackish‐grey silver chloride when exposed to the high voltage and current flow from the current drive circuit, induced by the change‐of‐state or switching of the ADC unit. Owing to the use of electrolyte‐gated *a*‐IGZO‐based TFTs, the entirely printed analog front‐end is found to be operable at a small supply voltage of 1–2 V, thereby making the all‐printed smart tag compatible with on‐chip, local energy sources such as micro‐supercapacitors and micro‐batteries.

## 2 RESULT AND DISCUSSION

### 2.1 Fabrication and electrical characterisation of electrolyte‐gated a‐IGZO TFTs and deep‐subthreshold operated depletion‐load type unipolar inverters

**FIGURE 1:**
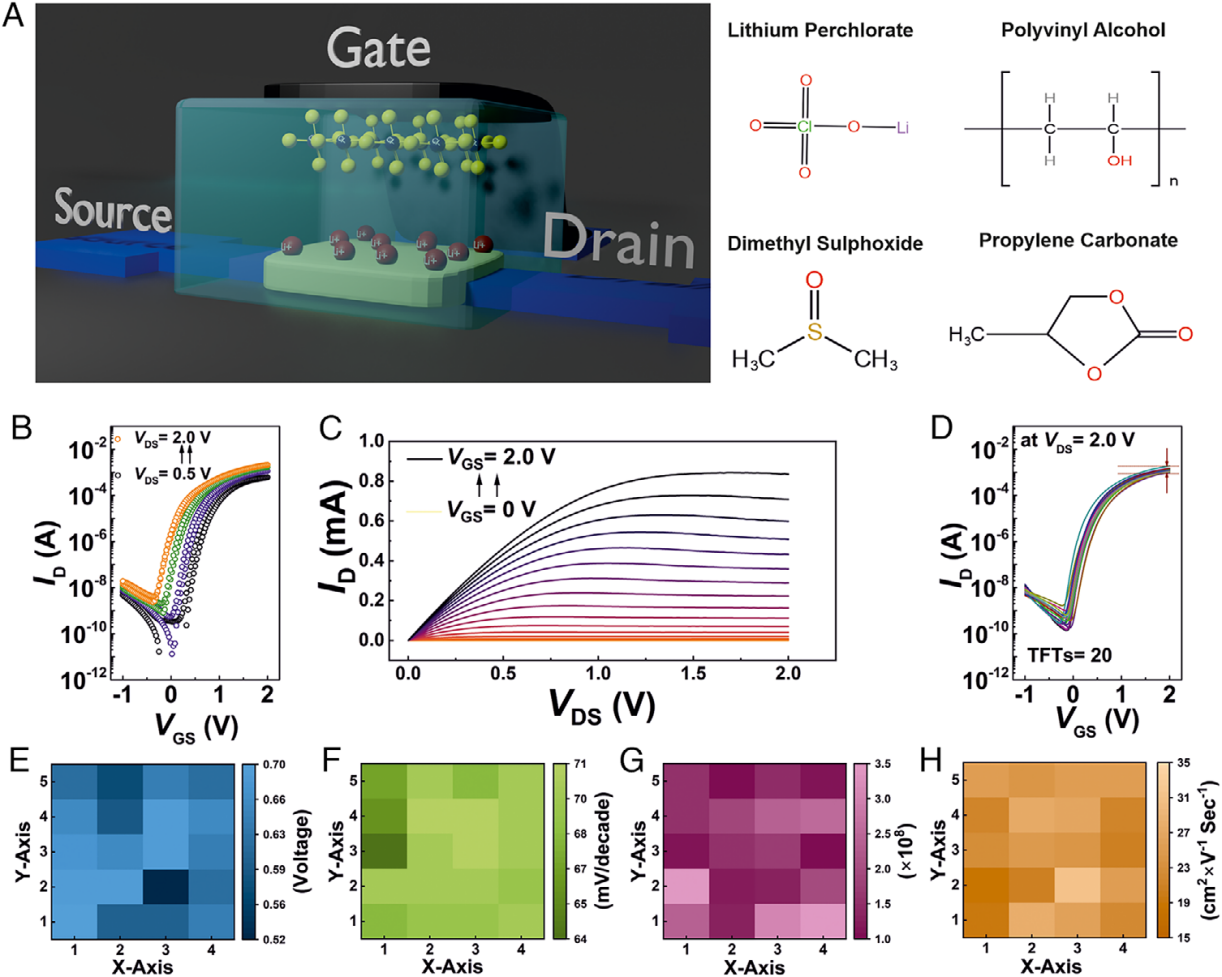
Fabrication and characterization of electrolyte‐gated *a*‐IGZO semiconductor based TFTs. (A) A schematic showing the *a*‐IGZO semiconductor channel electrolyte‐gated TFT (EG‐TFT) device and the constituents of the composite solid polymer electrolyte (CSPE). (B,C) The transfer and output characteristics of an archetypal EG‐TFT, respectively. (D) A statistics plot combining the transfer characteristics of 20 EG‐TFTs at *V*_DS _= 2 V, showing very low variability. (E–H) Statistical distribution of threshold voltage, subthreshold slope, on/off ratio, and linear mobility at *V*_DS _= 1 V, respectively, extracted from an array of 20 (5 × 4 array) EG‐TFTs.

The inkjet‐printed, electrolyte‐gated, *a*‐IGZO semiconductor channel TFTs have been fabricated on glass substrates. This has been followed by the subsequent printing of composite solid polymer electrolyte (CSPE)‐based gate insulator and poly(3,4‐ethylenedioxythiophene) polystyrene sulfonate (PEDOT:PSS) as the top gate electrode.^[^ ^46^, ^49^, ^57^, ^58^, ^59^ ^]^ A schematic of the printed bottom‐contact, top‐gate TFT device is shown in Figure 1A, and representative transfer and output characteristic curves are shown in Figure 1B,C, respectively. A statistics plot comprising the transfer characteristics of 20 TFTs (*W*/*L *= 20 µm/50 µm) is shown in Figure 1D that demonstrates high reproducibility and low variability in device performance, which is quite essential for reliable circuit operation. Here, the ON‐state drain current can be noted as 1.2 mA; this is a particularly high value for a *W*/*L* ratio of 0.4. It is only possible due to a conformal semiconductor‐electrolytic insulator interface, thereby largely increasing the gating efficiency.^[^ ^7^, ^60^, ^61^ ^]^ On the other hand, the large electric double layer (EDL) capacitance of the electrolytic insulator can induce strong band bending and rapidly reduce the ITO/*a*‐IGZO contact resistance within a small applied gate potential (≈0.5 V) to ensure semiconductor‐electrode ohmic contact formation.^[^ ^46^ ^]^ In effect, the recorded subthreshold slope (*S*) is found to be very close to Boltzmann's limit (60 mV decade^−1^) to support high transconductance efficiency. This contributes to the high signal gain in printed inverters and a large amplification ratio of the printed amplifiers. In addition, the selected large channel lengths (*L *= 50 µm) of the TFTs immune them from the channel‐length modulation effects, which leads to high output resistance (*r*_o_). In combination, the superior transconductance efficiency and the high output resistance are essential to obtain high intrinsic gain (*A*_i_), which is again mandatory for efficient, low‐power electronic circuits (Figure S2, Supporting Information). Here, the devices are found to exhibit a very high on/off ratio of >10^8^, a positive average threshold voltage of 0.6 V, and an average linear mobility of 24.3 cm^2 ^V^−1 ^s^−1^. In Figure 1E–H, the important performance parameters of 20 TFTs, printed in a 4 × 5 array, on an identical substrate are demonstrated; once again, low variability in the estimated threshold voltage, subthreshold slope, on–off ratio, and linear mobility (Table S2, Supporting Information) ensures that the printed TFT technology is matured enough to aim at complex circuit demonstration.

**FIGURE 2:**
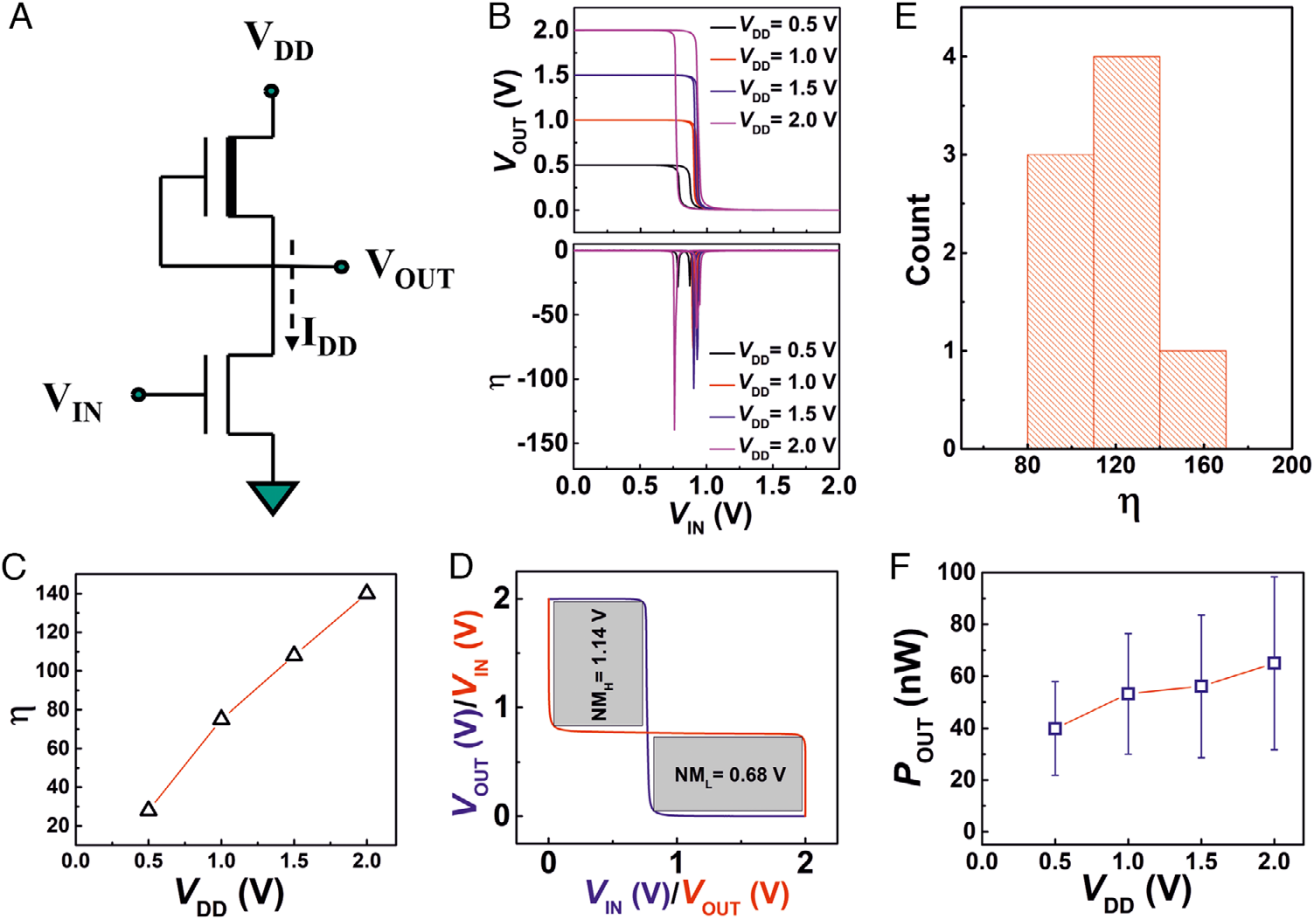
Deep‐subthreshold operated *a*‐IGZO TFT based depletion‐load type unipolar inverters. (A) A schematic of depletion‐load type inverter based on *a*‐IGZO EG‐TFTs, where the LOAD TFT is having *W*/*L *= 50 µm /20 µm and the DRIVE TFT is with *W*/*L *= 20 µm/50 µm. (B) The voltage transfer characteristics (VTC) and the respective voltage gain plot of a typical depletion‐load type inverter. (C) Variation of the signal gain of the inverter, with respect to *V*_DD_. (D) The estimated noise margin of the inverter, NM_H_ and NM_L_ for *V*_DD _= 2 V. (E) The statistics plot of the voltage gain for depletion‐load type inverter based on EG‐TFTs at *V*_DD _= 2 V. (F) The dynamic power consumption (*P*_OUT _= *V*_OUT_×*I*_DD_) of the depletion‐load type inverter with respect to the supply voltage, *V*_DD_.

Next, using the *a*‐IGZO TFTs, deep‐subthreshold operated unipolar pseudo‐CMOS inverters have been fabricated that show excellent noise immunity characteristics. The schematic of the inverter design is shown in Figure 2A. In this case, the DRIVE TFTs are fabricated with a device aspect ratio of *W*/*L *= 20 µm/50 µm, whereas the LOAD TFTs have been designed with *W*/*L = *50 µm/ 20 µm. The selected large variation in the device aspect ratio for the DRIVE and LOAD TFTs ensures that the DRIVE TFTs always operate in a deep‐subthreshold regime. A representative voltage transfer characteristic (VTC) of the depletion‐load type inverter is shown in Figure 2B. The inverter gain (*η*) is a function of the supply voltage; the correlation of *V*_DD_ with *η* for a particular inverter is shown in Figure 2C. Here, the maximum observed signal gain of the inverter is 140 at a supply voltage of 2 V; the statistics of maximum inverter gain for several inverters are shown in Figure 2D. It may be noted that the transition of the output voltage from the high‐state to the low‐state is very close to *V*_DD_/2, resulting in noise margin values that is, NM_H _= *V*_OH _− *V*_IH_ and NM_L _= *V*_IL_–*V*_OL_, both to be quite high, as shown in Figure 2E. On the other hand, despite being unipolar logic, owing to the deep‐subthreshold operation of the DRIVE TFTs, the dynamic power consumption, that is, *P*_OUT _= *V*_OUT _× *I*_DD_ of the devices is found to be very low, in the tens of nW range (Figure 2F). The dynamic power consumption of a representative device with observed signal gain at different *V*_DD_ values is shown in Figure S3, Supporting Information.

### 2.2 Common‐source and differential amplifiers

**FIGURE 3:**
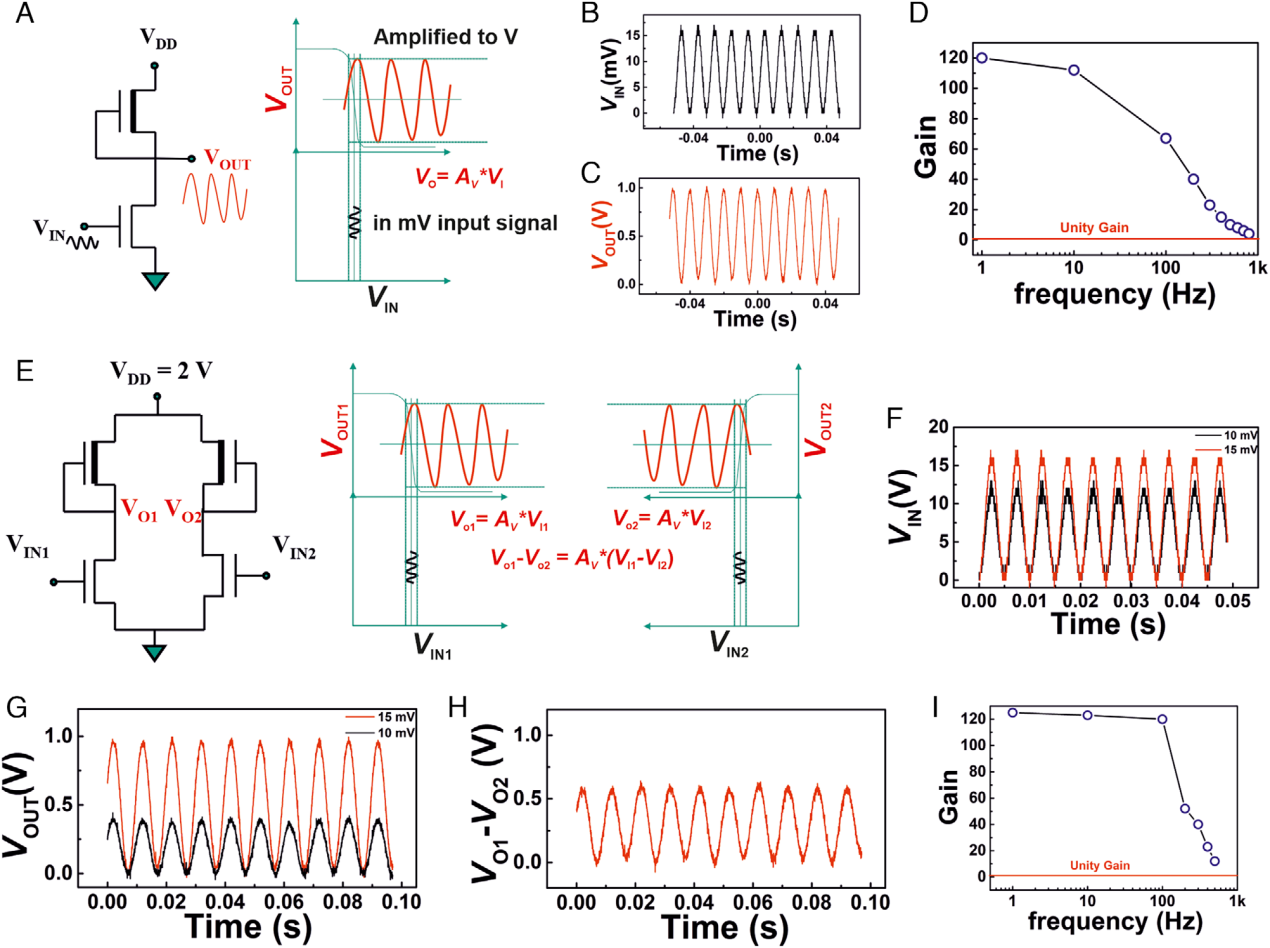
Deep‐subthreshold operated *a*‐IGZO TFT based common‐source and differenetial amplifiers. (A) A schematic of depletion load type inverter based on EG‐TFT, and the working principle of a common source amplifier. (B) A sinusoidal input voltage signal (*V*_IN_) of 15 mV at 100 Hz, which is fed to the common source amplifier. (C) The amplified sinusoidal output voltage signal (*V*_OUT_) of 1 V at 100 Hz, received from the common source amplifier. (D) The signal gain versus frequency plot of *a*‐IGZO semiconductor channel EG‐TFT derived depletion‐load type common source amplifier. (E) The schematic of a differential amplifier based on depletion‐load type inverters and the working principle of a differential amplifier. (F) Two different sinusoidal input voltage signals of 10 and 15 mV, fed into the differential amplifier; (G) the amplified sinusoidal output voltage signal of the two different sinusoidal input signals of 10 and 15 mV; (H) the difference of the two amplified output signal for the corresponding input sinusoidal signal of 10 and 15 mV. (I) The signal gain versus frequency plot for an *a*‐IGZO semiconductor channel EG‐TFT based differential amplifier.

A single inverter can also act as a common‐source amplifier, as shown in Figure 3A; when a very small AC (sinusoidal) signal, the value of which is placed at the maximum gain of the inverter, is superimposed on a DC bias, a large amplification of the input AC signal can be achieved. Figure 3B,C shows the common‐source amplifier operation with a depletion‐load type unipolar inverter; here, a peak‐to‐peak sinusoidal signal of 15 mV at 100 Hz has been superimposed on a DC bias of 0.35 V, and an amplification of the input AC signal to a peak‐to‐peak output of 1 V has been achieved. The signal gain versus input signal frequency is shown in Figure 3D. This infers that the amplification capacity of the electrolyte‐gated inverters is quite substantial at lower frequency values, and at higher frequencies, the amplification decreases to reach unity amplification at 1 kHz, which is a gain‐bandwidth product of nearly 1000. However, when such amplifiers are employed at sensor interfaces, the operation frequency of 100 Hz would typically be sufficient for all practical applications; for example, the high gain values at lower frequencies can be used to amplify even the ultra‐small electrophysiological signals at µV range to an easy‐to‐record mV level (Figure S5, Supporting Information).

Next, it is demonstrated that differential amplifiers may also be fabricated following an identical protocol; here, four TFTs are used, two DRIVE (*W*/*L *= 20 µm/50 µm) and two LOAD TFTs (*W*/*L *= 50 µm/20 µm) are connected to the common supply (*V*_DD_) and common ground (GND), respectively, as shown in Figure 3E. It operates in a similar manner as the common‐source amplifier the only difference is that the output signal from both the inverters, connected side‐by‐side, is considered, and differentiated to find out the resultant output signal. In effect, the differential amplifier produces an amplified output signal, which is proportional to the voltage difference of the two input signals (*V*_IN1_ and *V*_IN2_) applied to the respective inverters, as shown in Figure 3F. Notably, the differential amplifiers are used to magnify small noisy signal, where the differential input is used to eliminate the noise. Consequently, this circuit is also called a subtractor circuit.

Here, the electrolyte‐gated differential amplifier is characterized by two different amplitudes of sinusoidal input signal; that is, the inverters are provided with a peak‐to‐peak sinusoidal signal of 15 and 10 mV, respectively. The differential output voltage (*V*_D_) has sinusoidal oscillation of 0.6 V, which is an amplified version of the 5 mV differential input (*V*_IN1_ − *V*_IN2_) signal, as shown in Figure 3G–I. As the principle of operation of a differential amplifier is similar to that of a common‐source amplifier, the amplification in relation to the input signal frequency is expected to be identical. Indeed, a similar trend has been noted (Figure 3I), where up to the input signal frequency of 100 Hz, the high amplification ratio is maintained, and later a quick drop towards higher frequencies is observed. Here, a comparison of our amplifier performance with previous literature reports on solution‐processed amplifiers based on either organic semiconductors, single‐walled carbon nanotubes (SWCNTs), or amorphous oxides is summarized in Table 1.

**TABLE 1:** Comparison for our printed circuit elements that comprise the sensor patch with solution processed amplifiers and ADCs reported in the literature.

Semiconductor material	Fabrication process	Circuit elements	Operation voltage	Amplification	Citation
TIPS‐Pentacene	Screen printing	Differential amplifier and digital‐to‐analog converter (DAC)	60 V	22	[63]
Pentacene	Spin coating	Shift register	35 V		[64]
*p*‐type IDT‐BT and *n*‐type a‐IZO	Spin coating	Differential amplifier	5 V	–	[65]
SWCNT	Spin coating	Amplifier	2 V	68	[66]
SWCNT	Spin coating	D‐ flip flop	6 V	–	[67]
SWCNT	Spin coating	Amplifier	5 V	100	[68]
Pentacene	Spin coating	Differential amplifier	5 V	22	[69]
Pentacene	Spin coating	ADC	15 V	–	[36]
Pentacene	Spin coating	ADC	20 V	–	[35]
SWCNT/a‐IGZO	Spin coating	Amplifier	40 V	18	[70]
a‐IGZO	Spin coating	Op‐Amp	15 V	16	[71]
a‐IGZO	Inkjet printed	Complete sensor patch including amplifier, differential amplifier, ADC, visual demonstrator etc.	2 V	120	This work

### 2.3 Analog‐to‐digital converter (ADC) based on depletion‐load type inverters

**FIGURE 4:**
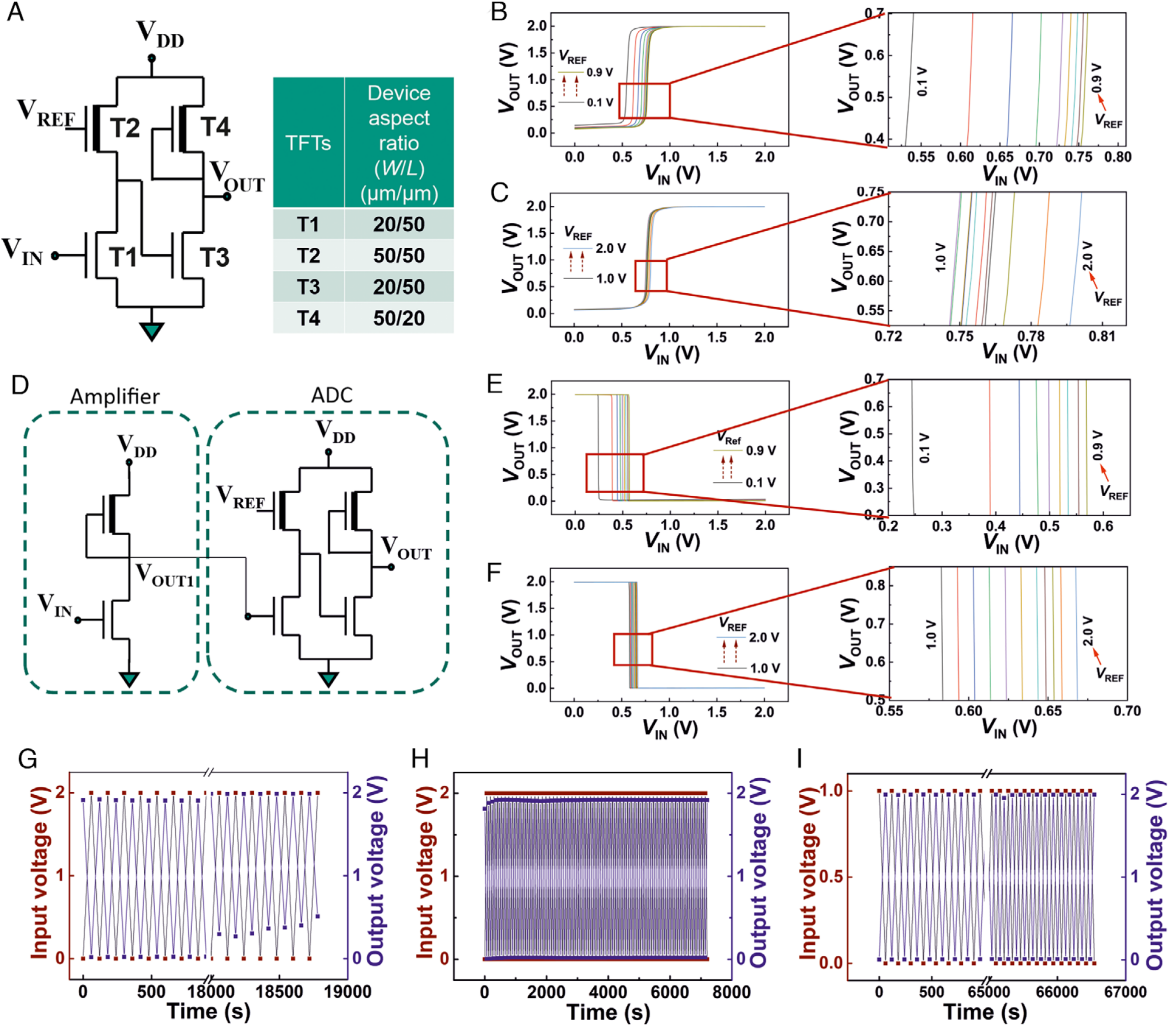
Electrical characterization of deep‐subthreshold operated *a*‐IGZO based common‐source amplifier and ADC circuit. (A) A schematic representation of the proposed 4 transistor, 1‐bit ADC circuit, with selected device aspect ratio of each EG‐TFTs, (B,C) VTC of the ADC with respect to the applied reference voltage (*V*_REF_). (D) The schematic of the proposed 4‐transistor, 1‐bit ADC, with a common source amplifier to amplify the input signal. (E,F) The VTC of the ADC with the common source amplifier with respect to the applied *V*_REF_, demonstrating a sharper transition of the states with an amplified input signal. (G) The recorded output voltage from the ADC with the common source amplifier circuit, at a constant supply volatge of *V*_DD _= 2 V, while the input voltage is swept between 0 to 2 V, and it shows a distortion in the output signal, after 18,000 s. (H) The same circuit demonstrated undistorted ADC output after 10 min of rest time. (I) Demonstration of undistorted 0 to 2 V digitalized output signal, even after 18 h of continuous biasing, when the input signal is swept between 0 to 1 V.

Analog‐to‐digital converter (ADC) is the key component of any communication system. It is required to convert the analog/continuous input signal into the digitalized output signal. Here, we demonstrate the feasibility of a fully‐printed smart sensor tag with printed sensors, common‐source/differential amplifiers, ADCs, and a current drive circuit that can be operated at an on‐chip energy source (micro‐supercapacitors or micro‐batteries) compatible with low voltage (1–2 V) values. The scheme of the entire printed sensor tag with an on‐chip power supply and visual recognition of the sensor signal is shown in Figure S6, Supporting Information. In this regard, at first, a simple ADC circuit design is proposed, which would be easy‐to‐print and offer stable performance with high reproducibility. The proposed ADC design is schematically shown in Figure 4A; initially, only a 1‐bit ADC unit is conceived and demonstrated, which would act as a switch and record a predefined threshold crossover of any selected sensor unit. The proposed ADC unit consists of four TFTs, in which, the device aspect ratio of the TFTs has been carefully chosen, as shown in Figure 4A. Here, the designed 1‐bit ADC unit has two stages (analogous to 2 inverters connected in series), where the DRIVE TFT of the first stage receives the input analog signal from the sensor, while the LOAD transistor is fed with a reference voltage (*V*_REF_), the value of which controls the position of switching of the ADC output with respect to the analog sensor signal, as shown in Figure 4B,C. Here, the applied *V*_REF_ determines the input voltage of the T3‐TFT and thereby the voltage distribution between the DRIVE and LOAD TFT at the second stage, and in turn, it controls the output voltage of the ADC unit with respect to the sensor signal. Therefore, the *V*_REF_ is the tool to control the predefined threshold point of the sensor signal, where the digitalized ADC output would switch from one state to the other. It may also be noted that initially, the change in *V*_REF_ from 0.1 to 0.9 V causes a large shift in the output voltage of the ADC with respect to the input sensor signal; however, beyond 0.9 V, the degree of the ADC output shift reduces considerably. This is actually related to the transfer characteristic of the T2‐TFT (Figure S7, Supporting Information); the shift in the ADC output is large when the transistor channel conductance is in the linear regime, and the shift becomes shallow when it enters the saturation region. The schematic of the ADC unit with a common‐source amplifier to amplify the sensor signal is shown in Figure 4D; with the addition of the amplifier before the ADC unit, the switching characteristics of the ADC output become substantially sharper, as shown in Figure 4E,F. At this point, in order to verify the environmental stability, and shelf‐life of the circuit elements, or the printed smart sensor patch, both the ADC unit and the amplifier plus ADC unit have been re‐measured after 1 month to record identical circuit performance, even when they have not been encapsulated (Figures S8 and S9, Supporting Information). Next, the common‐source amplifier and the ADC unit are tested together for their switching reliability or stability. Initially, the amplifier alongside the ADC unit is supplied with an input signal sweep between 0 and 2 V, with a constant supply voltage, *V*_DD _= 2 V. The ADC in response shows digitalized, undistorted output data between 0 and 2 V, up to 5 h, after which the ADC output is noted to be distorted and not completely going back to 0 V (Figure 4G). However, after 10 min of idle time, the ADC unit again demonstrates complete switching, as shown in Figure 4H. On the other hand, when the input voltage sweep is reduced to 0 to 1 V, the switching of the ADC unit with respect to the input signal is observed to remain completely unaltered for more than 18 h (Figure 4I).

**FIGURE 5:**
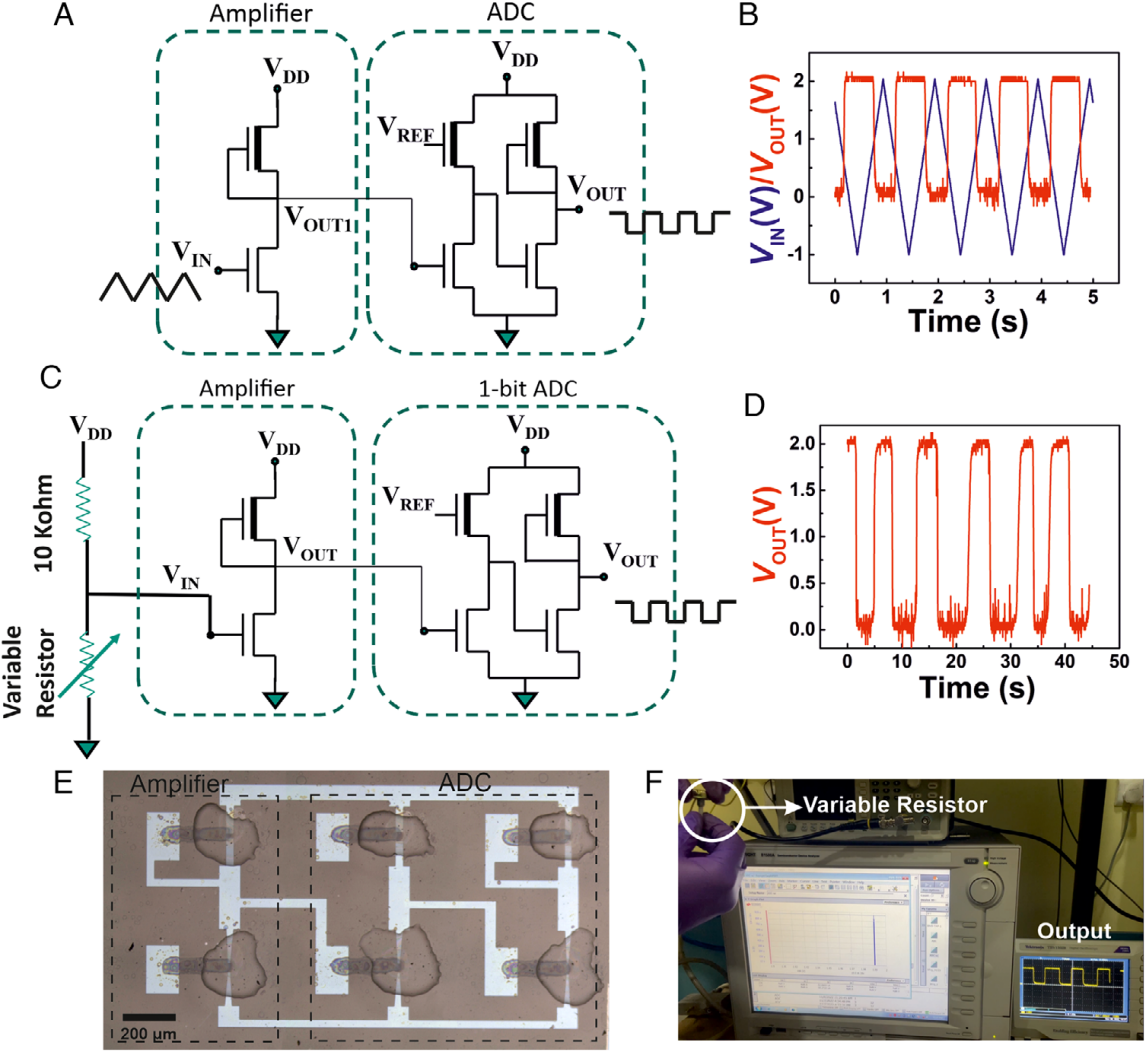
Dynamic response from a variable resistor to control the ADC switching. (A) A schematic of the proposed 1‐bit ADC along with the common source amplifier circuit, showing analog input signal to convert to digitalized output signal. (B) The AC measurement of the ADC, where a triangular input signal is successfully converted to square output. (C) A schematic of the proposed ADC connected to a voltage divider circuit with a variable resistor. (D) The output voltage waveform of the ADC in response to a change in the resistance of the variable resistor. (E) An optical image of the printed ADC along with the single inveter amplifier circuit. (F) An optical image of the realtime experiment, where the output voltage waveform is being altered continuously with the change in resistance at the inverter input by manually changing the resistance of the variable resistor (see Movie S1, Supporting Information).

The analog‐to‐digital conversion of a triangular waveform input to a square‐wave output is shown in Figure 5A,B; with an applied triangular input of 1 V, the output voltage swings from 2 to 0 V when the input voltage rises above 0.6 V, and it swings back to 2 V when the input voltage falls below 0.6 V (Figure 5B), thereby demonstrating a clear analog‐to‐digital signal conversion. The behaviour at the chosen operation frequency of 30 Hz is shown in Figure S10, Supporting Information. Next, a voltage divider circuit that constitutes a constant resistor (10 kΩ) and a variable resistor in series is connected before the amplifier unit, where the constant resistor is connected to the supply voltage, *V*_DD_, and the variable resistor/rheostat, which controls the voltage to be fed to the amplifier unit, is connected to the ground, as shown in Figure 5C. The entire arrangement must respect one important precondition: that the resistance connected across the input of the amplifier unit cannot be greater than the gate‐source resistance of the receiving transistor. The maximum gate current of the electrolyte‐gated TFTs typically varies between 5 and 10 nA, as shown in Figure S11, Supporting Information. Therefore, it is safe to have the series resistance value of the voltage divider circuit up to 10 MOhm. Here, the output voltage of the ADC unit is measured, while the input voltage to the amplifier is controlled by the variable resistor, within a particular voltage range. Figure 5D shows the digitalized output voltage. Furthermore, it is to be noted that the value of variable resistance changes with respect to the applied reference voltage in the ADC unit, as shown in Table S3, Supporting Information. An optical viewgraph of the amplifier alongside the ADC unit is shown in Figure 5E. On the other hand, a video of the live experiment is provided in Movie S1, Supporting Information; a still image of the video showing the ADC output voltage variation in response to the resistance modulation in the variable resistor is shown in Figure 5F.

### 2.4 ADC circuit integrated with temperature sensor

**FIGURE 6:**
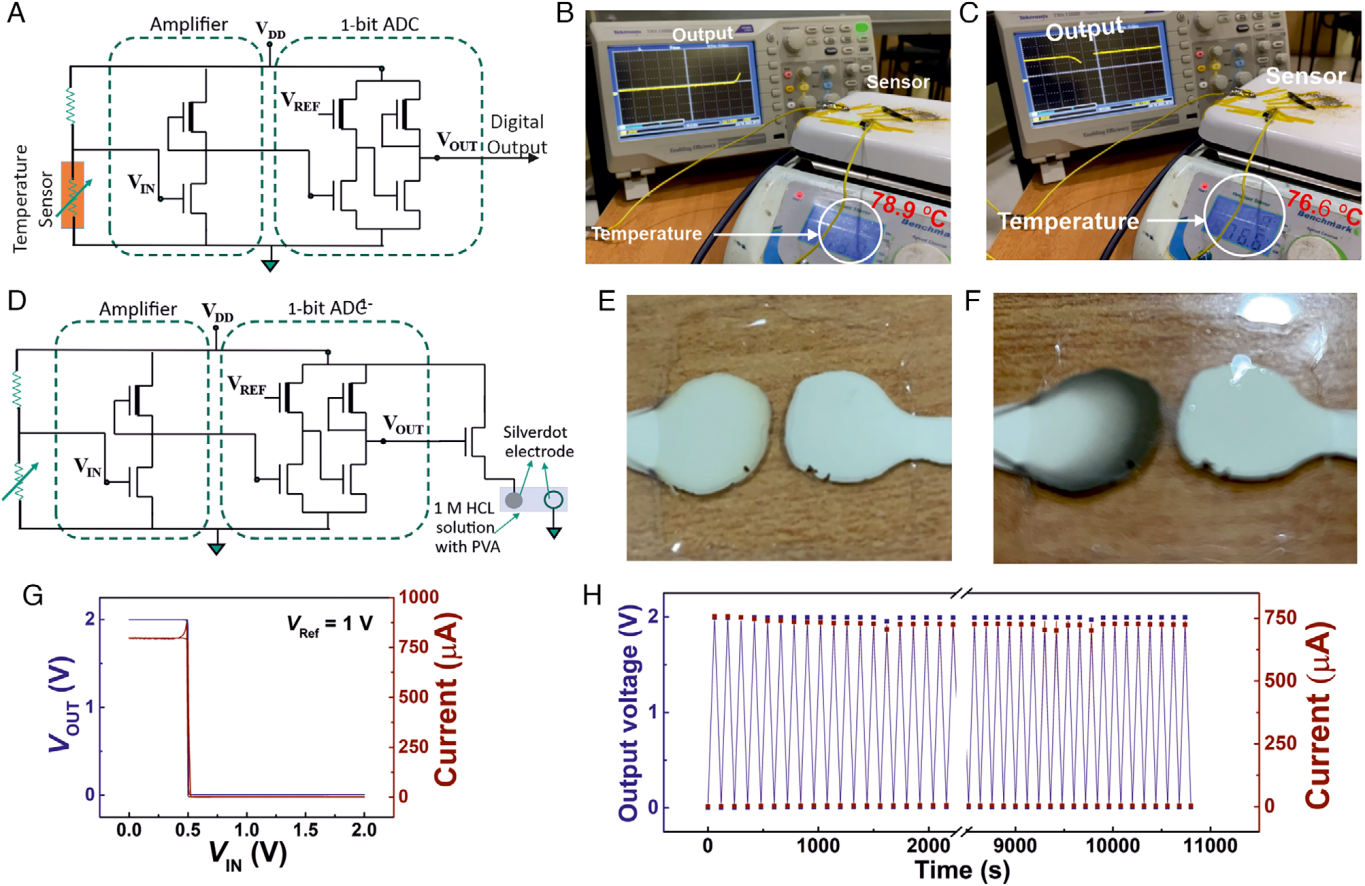
Printed readout electronics interface with a temperature sensor and demonstration of the ADC switching with a visual recognition unit. (A) A schematic of the proposed ADC with the common source amplifier circuit, where the input at the amplifier is from a Mxene based temperature sensor. (B) The output voltage of the ADC switches from 0 to 2 V, with respect to the change in resistance of the temperature sensor at a preset temperature of 77°C, defined by the *V*_REF_ set at 1 V. (C) The output voltage switches back from 2 to 0 V, when the temperature sensor is cooled down below 77°C. (D) A schematic of the proposed ADC circuit, which is fed by a voltage divider circuit and the output of the ADC being connected to a current drive circuit for a high current output. (E,F) Demonstration of the visual recognition of the ADC switching with two printed silver electrodes with HCl based solid electrolyte. (G) The VTC of the current drive circuit with the output current switch, when the ADC output varies between 0 to 2 V. (H) The current drive circuit under continuous dynamic switching, the output voltage of the ADC and the measured current from the current drive circuit is plotted with respect to time, when the input voltage has been swept between 0 to 1 V, with a constant *V*_REF_ of 1 V, for more than 3 h of biasing.

After observing the performance of the amplifier and the ADC unit with the voltage divider circuit, it has been possible to anticipate that the circuit would work with an actual resistive sensor as well. A resistive sensor is an active material placed between two metal electrodes, whose resistance changes with the change in an external stimulus, which can be anything between temperature, humidity, pressure, proximity, UV, visible light, IR, gas, volatile organic species (VOCs), chemicals, metal ions, biological molecules etc. The change in the sensor unit would change the voltage drop across it, which in turn varies the intermediate voltage of the voltage divider circuit, the voltage that is fed to the common‐source amplifier (Figure S6, Supporting Information). In order to demonstrate the 1‐bit ADC trigger by a real sensor unit, a temperature sensor fabricated with MXene (Ti_3_C_2_T*_x_*) 2D metal sheets in combination with polyvinyl alcohol (PVA) and polyvinyl pyrolidene (PVP) has been used, as shown in Figure 6A. It is observed that the state of the ADC output voltage changes from 0 to 2 V when the temperature experienced by the MXene‐based temperature sensor exceeds 77°C, and it reverses to 0 V when the experienced temperature by the sensor unit reduces below 77°C, as shown in Figure 6B,C and Movie S2, Supporting Information. The observed few degree Celsius hysteresis is primarily due to the temperature sensor itself, as shown in Figure S15d, Supporting Information. The temperature at which the ADC output switches (from 0 to 2 V) can be adjusted for the same sensor either by adjusting the resistor connected in series with the sensor unit, thereby changing the input voltage at the common‐source amplifier, or by changing the value of *V*_REF_ in the ADC unit. This has been demonstrated with the same sensor unit, where the temperature set‐point for the ADC switching has been altered to 32°C, as shown in Figure S16 and Movie S3, Supporting Information. Considering the above experimental results, one can be assertive that the printed sensor‐amplifier‐ADC combination is working as a switch that can easily detect a certain lower or upper threshold value crossover of any sensor unit. At the next step, multi‐bit ADC units may also be fabricated to further discretize the sensor output to multiple ranges (Figure S17, Supporting Information).

### 2.5 ADC unit alongside the current drive circuit for high current applications

In order to easily comprehend the switching of the ADC unit at the user end, it is essential to have a specific work done that would help to visualize the event of the digital switching. This would require a high power output, whereas the ADC output is purely a potential variation with very low current flowing through the inverter‐like units, especially when the DRIVE TFTs are always near their off‐state. Therefore, in order to ensure an easy visualization of the ADC switching, a current drive circuit must be connected, after the ADC unit. This current drive circuit may simply be a few high‐current oxide TFTs placed in parallel, whose drain electrodes are connected to the supply voltage, and the gate electrodes are connected to the output of the ADC unit, as shown in Figure S6, Supporting Information. When the ADC output is 0 V, these current drive circuit TFTs are in their Off‐state, however, when the ADC output is 2 V, the large positive gate voltage on these *n*‐type oxide TFTs would ensure that they are in their ON‐state, and therefore a large current would flow through these TFTs. In the present study, the current drive circuit consists of only one TFT; as the printed oxide TFTs can easily carry milliamperes of current, it has been found sufficient for the demonstrator that is shown in this study (Figure 6D–F). Here, the source electrode of the current drive circuit TFT is connected to the visual recognition patch (Figure 6D). The visual recognition patch is comprised of two printed silver circles and a printed aqueous solid polymer electrolyte based on 3 wt% of PVA and 1 M HCl, which covers the silver electrodes. While one of the two printed silver circles is connected to the current drive circuit TFT, the other one is connected to the common ground (Figure 6D). When the ADC is in its off‐state, the current drive circuit TFT is also in its off‐state, thereby the entire supply voltage (*V*_DD_) drops across that current drive circuit TFT, and the printed silver circle receives 0 V. Therefore, there is no potential difference between the silver electrodes, and hence both of them appear shiny and silvery in colour. However, when the ADC output changes to 2 V, the current drive circuit TFT switches on, with minimal resistance and a potential drop across it; this also allows a large current to flow. In this situation, a potential difference of 2 V is applied across the printed silver circles, which leads to AgCl formation at the silver circle that is at a higher potential. The AgCl being dark grey in colour, the silver electrode that is connected to the current drive circuit turns blackish grey, thereby providing a clear visual recognition of the ADC switching event (Figure 6E,F; Movie S4, Supporting Information).

The VTC plot of the entire circuit is shown in Figure 6G, where the current passing through the circuit is high, that is, around 800 µA, when the output voltage of the ADC unit is high (2 V), and the current passing through the circuit is negligible when the output voltage of the ADC unit is low. Here, with a constant supply voltage of 2 V, when the input voltage of the amplifier is switched continuously between 0 and 1 V, not only does the output voltage of the ADC change between 2 and 0 V, but the output current of the current drive circuit also changes in phase with the ADC switching (Figure 6H). The circuit performs well over 3 h without any deterioration in its performance, as shown in Figure 6H and it is believed that the circuit may perform in a similar manner for more than 18 h, as has been the case for the ADC unit with the input voltage varying between 0 and 1 V (Figure 4I). Summarizing it up, in the present study, the idea has been to utilize the voltage‐controlled current source mode circuit to identify the pre‐defined sensor signal crossover, or the ADC switching, with the help of a visual recognition unit, which may either be electrochromic or LED lighting, or a chemical change‐induced variation in colour of a material etc. On the other hand, being a one‐time event, such printed sensor tags with read‐out electronics may also be used as a high‐tech, and difficult‐to‐replicate, anti‐counterfeit tag, where the first‐time exposure of the sensor unit to some specific stimulant, for example, light or air (oxygen or humidity), or a specific chemical substance can be used to trigger the change of state of the ADC unit, which with the help of the visual recognition unit can ensure the authenticity of a particular product. At the end, it is also interesting to note that the entire printed readout electronics are optically transparent and show above 90% transparency over the entire visible spectrum (as shown in Figure S18, Supporting Information).

## 3 CONCLUSION

In summary, a fully‐printed smart sensor tag with readout electronics is presented. The analog front‐end uses electrolyte‐gated oxide TFTs and *n*‐type unipolar logics; however, owing to the deep subthreshold operation of all the DRIVE TFTs, the power consumption of the circuits remains low to only a few nW levels. On the other hand, the depletion‐load type inverter design offers a high signal gain of 140 at a supply voltage of 2 V and superior noise immunity. Next, the common‐source and differential amplifiers are demonstrated with an amplification ratio of 120 and a fairly steady amplification ratio up to 100 Hz, which is certainly sufficient to be used at sensor front‐end readout electronics. This follows an easy‐to‐realize, four transistors 1‐bit ADC design, which can successfully digitalize the input analog signal. Next, with the help of a current drive circuit and visual demonstration unit, it has been possible to demonstrate a fully‐printed smart sensor tag, which can identify the crossover of a predefined threshold of the sensor and provide easy recognition of the event. It may be foreseen that such fully printed sensor tags can have a wide range of applications, for example, at industrial premises, for food and drug safety, as biosensors and medical diagnostic kits, for soil tests, or for various similar applications where an inexpensive and large volume of reliable smart sensor tags are required.

## 4 EXPERIMENTAL METHODS

### 4.1 Semiconductor ink preparation

The inkjet printable 0.03 M *a*‐IGZO semiconductor precursor ink was prepared (with an atomic ratio of In:Zn:Ga = 70:20:10) by dissolving indium (III) nitrate hydrate ([In(NO_3_)_3_·*x*H_2_O], 99.99%, trace metal basis), gallium nitrate hydrate (Ga(NO_3_)_3_·*x*H_2_O, 99.99%, trace metal basis), and zinc acetate ((CH_3_CO_2_)_2 _Zn, 99.99%, trace metal basis) in deionised water, ethanol (C_2_H_5_OH, 99.99%), and ethylene glycol (EG, C_2_H_6_O_2_, 99.99%), maintaining a (V/V) ratio of 45:40:15. Here, all the chemicals were procured from Sigma‐Aldrich Chemie GmbH and used as received without further purification.

### 4.2 Preparation of composite solid polymer electrolyte (CSPE) ink

In order to prepare the composite solid polymer electrolyte (CSPE), the 0.3 g of synthetic polymer, poly(vinyl alcohol) (PVA), was dissolved in 5 mL of DMSO, at 90°C for about 1 h. The supporting electrolyte/salt, 0.07 g of lithium perchlorate (LiClO_4_) was dissolved in 1 mL of plasticizer, propylene carbonate (PC), at room temperature. After 1 h of stirring, both solutions were mixed together and stirred for another 12 h at room temperature in order to obtain a completely homogeneous CSPE solution.^[^ ^60^, ^62^ ^]^ All the chemicals were procured from Sigma‐Aldrich Chemie GmbH as used as received.

### 4.3 Structural and morphological characterization

The structural characterization of the *a*‐IGZO films was carried out using Rigaku SmartLab grazing incidence X‐ray diffractometer with Cu‐K_α_ X‐ray source (40 kV, 30 mA) and a constant grazing incidence angle of 0.5°. The morphological characterization and energy dispersive X‐ray (EDX) analysis of the printed thin films were carried out using Ultra55 FE‐SEM (Karl Zeiss).

### 4.4 Device fabrication

The thin‐film transistor design is shown in Figure 1A. The source and drain electrodes were defined by lithographically patterned ITO electrodes. The inkjet printing was carried out using Dimatix 2831 functional materials printer. The used 0.03 M *a*‐IGZO (In:Zn:Ga = 70:20:10) precursor ink contained 15 vol% ethylene glycol, which was added to ensure homogeneous, predominantly amorphous film formation. The printing of the *a*‐IGZO semiconductor ink was followed by immediate pre‐heating at 90°C and a subsequent annealing step at 350°C for 1 h. Next, printing of the CSPE ink as the gate insulator and the PEDOT:PSS ink as the top gate electrode was carried out on the fabricated semiconductor layer in order to complete the device fabrication process. The detailed printing parameters for each ink, that is, semiconductor, CSPE, and PEDOT:PSS inks are summarized in Table S1, Supporting Information. The unipolar NMOS depletion‐load inverters, common‐source amplifiers (*V*_OUT _= *A*×*V*_IN_), differential amplifiers (*V*_D _= *A*×(*V*_IN1_ − *V*_IN2_), where *V*_D_ is differential voltage, *A* is signal gain, *V*_IN1_ and *V*_IN2_ are input voltages), analog‐to‐digital converters (ADCs), and the current drive circuits were printed in an identical manner, comprising *a*‐IGZO TFTs of different aspect ratios, as mentioned in the relevant sections. Notably, prior to printing, each and every ink was filtered through a 0.2 µm polyimide‐based hydrophilic filter in order to avoid nozzle clogging and irregular printed patterns.

### 4.5 Electrical characterization

All the electrical measurements were carried out at room temperature and in ambient conditions. The electrical characterization was performed using a semiconductor parameter analyser (KEYSIGHT B1500A) connected to a MicroXact SPS1000‐15 DC probe station. For the AC measurements, a function generator (TEKTRONIX AFG1022) was used as the pulse/waveform generator at the input, and a digital oscilloscope (TEKTRONIX TBS1102B) was used to record the output voltage; in this case, the semiconductor parameter analyser (KEYSIGHT B1500A) was used to apply the constant supply/drive voltage, *V*_DD_. Next, the temperature sensor was integrated with the printed circuit comprising the inverter and ADC, as shown in Figure 6A–C, and the response was measured using the oscilloscope.

## AUTHOR CONTRIBUTIONS

Jyoti Ranjan Pradhan and Subho Dasgupta conceived the idea and designed the experiments; Jyoti Ranjan Pradhan fabricated the printed EG‐TFTs and circuits, measured the devices, and performed most of the characterization and data analysis with the supervision of Subho Dasgupta. Sushree Sangita Priyadarsini synthesized the Mxene ink, fabricated the temperature sensor, and performed its characterization. Manvendra Singh and Sanjana R. Nibgoor demonstrated the compatibility of the printed read‐out electronics, with the ARDUINO and ESP32 board (hybrid electronic). All authors contributed in the manuscript preparation.

## CONFLICT OF INTEREST STATEMENT

The authors declare no conflicts of interest.

## Supporting information

Supporting Information

Supporting Information

Supporting Information

Supporting Information

Supporting Information

Supporting Information

Supporting Information

## Data Availability

The data that support the findings of this study are available from the corresponding author upon reasonable request.
