# Supplementary material for: Oxide semiconductor based deep‐subthreshold operated read‐out electronics for all‐printed smart sensor patches
Source: Exploration (Beijing). 2024 May 15;5(1):20230167. doi: 10.1002/EXP.20230167 (PMC11875446; doi:10.1002/EXP.20230167)
Supplement: Supplementary file 1 — Supporting Information [file EXP2-5-20230167-s005.pdf]

Supporting Information

Oxide semiconductor based deep-subthreshold operated read-out electronics for all-printed smart sensor patch

*Jyoti Ranjan Pradhan, Sushree Sangita Priyadarsini, Sanjana R. Nibgoor, Manvendra Singh, Subho Dasgupta**

Department of Materials Engineering, Indian Institute of Science (IISc), C V Raman Avenue, Bangalore: 560012, Karnataka, India.

Contents:

A) XRD and SEM of the printed *a*-IGZO films.

B) Important device parameters extracted from electrolyte-gated TFTs (EG-TFTs) based on 0.03 M *a*-IGZO (In:70, Zn:20, Ga:10) precursor ink with 10 at.% gallium content.

C) Estimation of accurate linear mobility.

D) Dynamic power consumption of the depletion-load type *a*-IGZO-based NMOS inverters.

E) Noise margin estimation for the depletion-load type *a*-IGZO-based NMOS inverters.

F) The electrophysiological measurement with EG-TFTs based depletion-load type inverters.

G) A schematic showing the entire smart sensor patch with on-chip power source, sensor, amplifier, ADC, the current drive circuit, and the visual recognition unit.

H) The transfer and output characteristics of the EG-TFTs used in the ADC unit, fabricated with 0.03 M *a*-IGZO precursor ink with W/L= 50 µm/ 50 µm.

I) The voltage transfer characteristics (VTC) of the *a*-IGZO EG-TFT based analog-to-digital converter (ADC), re-measured after 1 month.

J) The VTC curve of the ADC alongside an amplifier, re-measured after 1 month.

K) The dynamic response of the ADC alongside the amplifier with a triangular analog input.

L) The gate current of a typical *a*-IGZO based EG-TFT device.

M) The printed ADC with amplifier circuit interfacing with the real world: connected to ARDUINO and ESP32 wireless board.

N) Synthesis of Mxene and Mxene-based temperature sensor.

O) Temperature sensor measurement with printed circuit set at critical temperature of 32 °C.

P) A schematic of a multi-bit ADC circuit.

Q) UV-visible spectroscopy of the device.

A) XRD and SEM of the printed *a*-IGZO films.

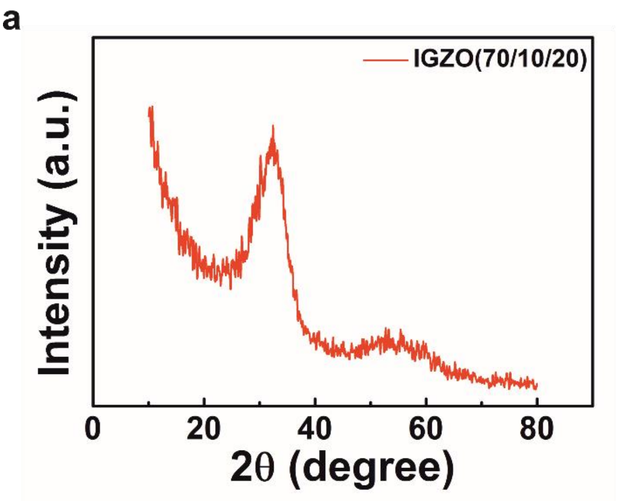

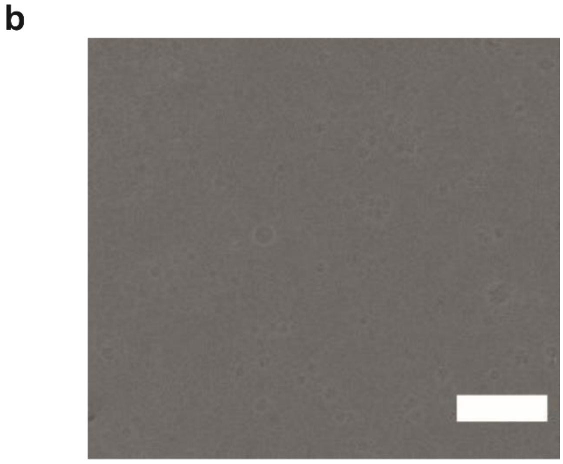

Figure S1: (a) Grazing incidence X-ray diffraction (GIXRD) pattern of the *a*-IGZO (In: 70, Ga: 10, Zn: 20) semiconductor film annealed at 350 °C; (b) Scanning electron micrograph of the a-IGZO film showing featureless amorphous morphology; the scale bar measures 500 nm.

The grazing incidence X-ray diffraction (GIXRD) pattern of 0.03 M *a*-IGZO (10 at% Ga), showing a predominantly amorphous structure (shown in Figure S1a); the very broad peak at $2\theta = 31°$ indicates that a fraction of phase separation induced small crystallites of indium oxide may be present there. The scanning electron micrograph confirms predominant amorphous nature of the semiconductor material, with entirely featureless amorphous-like microstructures (Figure S1b).

Table S1: Printing parameters of components used for the fabrication of EG-TFTs.

Inks	Platen temperature (°C)	Cartridge temperature (°C)	Meniscus set point	Cartridge voltage (V)	Drop spacing (µm)	No. of layers
a-IGZO semiconductor	30	25	3	40	5	2
CSPE	35	35	3	40	10	12
PEDOT:PSS	25	25	2	40	10	2

B) Important device parameters extracted from electrolyte-gated TFTs (EG-TFTs) based on 0.03 M *a*-IGZO (In:70, Zn:20, Ga:10) precursor ink with 10 at.% gallium content.

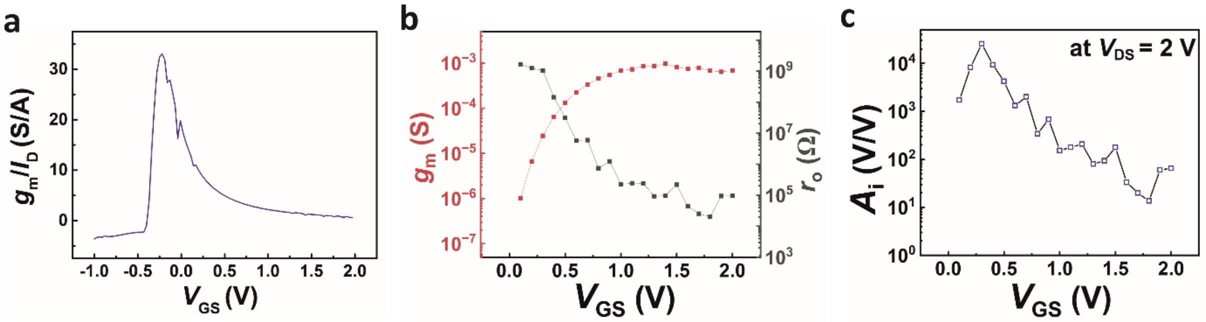

Figure S2: (a) Transconductance efficiency of a representative electrolyte-gated *a*-IGZO based TFT; (b) experimental values of transconductance (g_m) and output resistance (r_o= $\partial V_{DS}/\partial I_D$) with respect to V_{GS} at supply voltage, V_{DS}= 2 V; (c) calculated intrinsic gain (A_i) of the TFTs, measured at V_{DS}= 2 V.

The g_m/I_D vs. V_{GS} plot is used to estimate the transconductance efficiency; this demonstrates how efficietly the device can convert the drain current (I_D) into equivalent transconductance value (shown in Figure S2a). It is primarily determined by the TFT's subthreshold slope. It is worth mentioning that the transconductance efficiency drops dramatically above the threshold voltage as the subthreshold slope value increases sharply. However, below the threshold voltage, the ratio is high due to the lower value of subthreshold slope. Consequently, when the device/ TFT operates below the threshold voltage or in the deep-subthreshold region, the transconductance efficiency can be high, resulting in low power consumption for a circuit when operated at the deep subthreshold regime. On the other hand, the intrinsic gain (A_i= $g_m \times r_o$) is an important parameter of the device, which depends on two factors, i.e., transconductance (g_m) and output resistance (r_o). The g_m represents the device's transconductance, and the r_o represents the contact resistance between the semiconductor and the drive (source/drain) electrodes. The ITO passive (source/drain electrode) electrodes create a low barrier height Schottky contact with the *a*-IGZO semiconductor layer. However, the electrolytic insulator can easily tune the barrier formed between the drive (source/ drain) electrodes and the semiconductor layer due to its high capacitance value. Simultaneously, the use of a longer channel length (50 µm) reduces the drain to source drive field, resulting in proper saturation in drain current with respect to drain voltage at a constant gate voltage, resulting in a large output resistance, which ultimately provides a high intrinsic gain of the device (shown in Figure S2b-c).

C) Estimation of accurate linear mobility.

The linear mobility is typically estimated using the following relationship:

$$\mu_{lin} = \frac{L}{W\, C_{DL}\, V_{DS}} \left(\frac{\partial I_D}{\partial V_{GS}}\right) \tag{1}$$

where, L and W represent the transistor channel length and width, C_{DL}, the specific electric charge double layer capacitance, V_{DS}, the applied drain voltage (1.0 V has been considered for the calculation of μ_{lin}), I_D and V_{GS}, the drain current and gate voltage in the linear regime, respectively. In order to estimate the device mobility accurately, it is important to compute the C_{DL}, capacitance value accurately. Here, an accurate estimation of the same has been carried out for the oxide semiconductor EG-TFT device and it is estimated to be 14.2 µFcm^{-2}.[1,2]

However, the error in mobility calculation may also appear from an erroneous estimation of slope of the transfer curve, $\frac{\partial I_D}{\partial V_{GS}}$. In order to avoid such mobility overestimation arising from consideration of higher than slope of $\frac{\partial I_D}{\partial V_{GS}}$ n, it has recently been proposed that one may measure the slope of the entire transfer curve and estimate a reliability factor as follows:[3]

$$r_{lin} = \left[\left[\frac{|I_D|^{max} - |I_D|^0}{|V_{GS}|^{max}}\right] \Big/ \left[\left|\frac{\partial I_D}{\partial V_{GS}}\right|\right]_{\text{claimed}}\right] \times 100\% \tag{2}$$

where, r_{lin}, the reliability factor, $|I_D|^0$, the drain current at zero gate voltage; $|I_D|^{max}$, the drain current at $|V_{GS}|^{max}$, and $\left[\left|\frac{\partial I_D}{\partial V_{GS}}\right|\right]_{\text{claimed}}$, the slope of the I_D vs. V_{GS} plot that has been used for the mobility estimation. However, this equation is relevant for TFTs with threshold voltage value close to zero volts. Therefore, the above equation can be modified as:

$$r_{lin} = \left[\left[\frac{|I_D|^{max} - |I_D|^{V_T}}{|V_{GS}^{max} - V_T|}\right] \Big/ \left[\left|\frac{\partial I_D}{\partial V_{GS}}\right|\right]_{\text{claimed}}\right] \times 100\% \tag{3}$$

and then, the revised linear mobility ($\mu_{lin,actual}$) can be estimated as:

$$\mu_{lin,actual} = r_{lin} \times \mu_{lin,estimated} \tag{4}$$

Here, the reliability factor is estimated and has been multiplied to the $\mu_{lin,estimated}$ to obtain $\mu_{lin,actual.}$ Notably, the estimated reliability factor has typically been within the range of 0.6-0.7.

Here, the estimated linear mobility requires another corrective step, which is associated to the small *W*/*L* ratio and the printing of the semiconductor channel layer somewhat larger than the actual channel dimension results in stray currents beyond the device dimensions used for the mobility estimation (Equation 1). In order to mitigate this problem we assume a complete coverage of the semiconductor material over the entire substrate, certainly this would lead to an overestimation of the correction factor and an underestimation of the calculated mobility. Following a previous report, we estimate a correction factor of 2.8 for the device dimensions that we have used.[4] After consideration of all the correction factors stated above that can take

care of every possible overestimation in the calculated mobility value, we compute the linear mobility of the *a*-IGZO based EG-FETs to be 24±3 $cm^2V^{-1}s^{-1}$.

Considering a single device among all the devices, the device has estimated mobility value of 114 $cm^2V^{-1}s^{-1}$. With consideration of reliability factor r_{lin} of 0.66 (from equation 3), the $\mu_{lin,actual}$ is calculated to be 75 $cm^2V^{-1}s^{-1}$ which is still considered to be overestimation due to the stray currents associated to small *W*/*L* ratio been used in the device fabrication. So, to avoid the overestimation, here a correction factor of 2.8 has been used. By considering the above correction factor, the linear mobility is found to be 26.7 $cm^2V^{-1}s^{-1}$, which is quite approximately near to the linear mobility value calculated for higher *W*/*L* based devices in our previously reported literature.

Table S2: Important electrical parameters of the fabricated *a*-IGZO based EG-TFTs.

**Device aspect ratio (*W*/*L*) (µm/ µm)**	**$I_{D,ON}$ /W (µA/ µm)**	**g_m/W (µS/ µm)**	**V_t (V)**	**SS (mV/decade)**	**Linear mobility (cm^2/Vs)**	**On/Off ratio**
20/50	62.7±10	80.6±22	0.63±0.04	69.1±1.7	24±3	>10^8

D) Dynamic power consumption of the depletion-load type *a*-IGZO-based NMOS inverters.

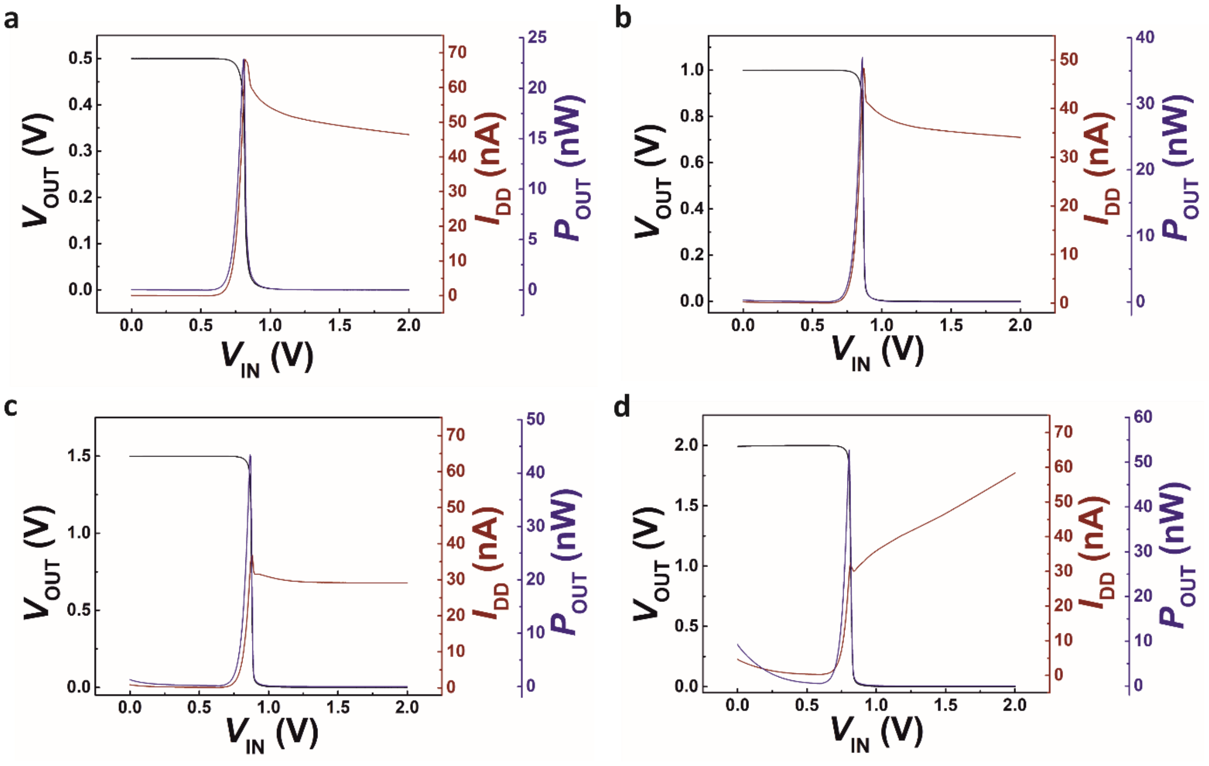

Figure S3: (a-d) Dynamic power consumption of the depletion-load type inverter with respect to each supply voltage V_{DD} from 0.5 V to 2 V, with a voltage step size of 0.5 V.

E) Noise margin estimation for the depletion-load type *a*-IGZO-based NMOS inverters.

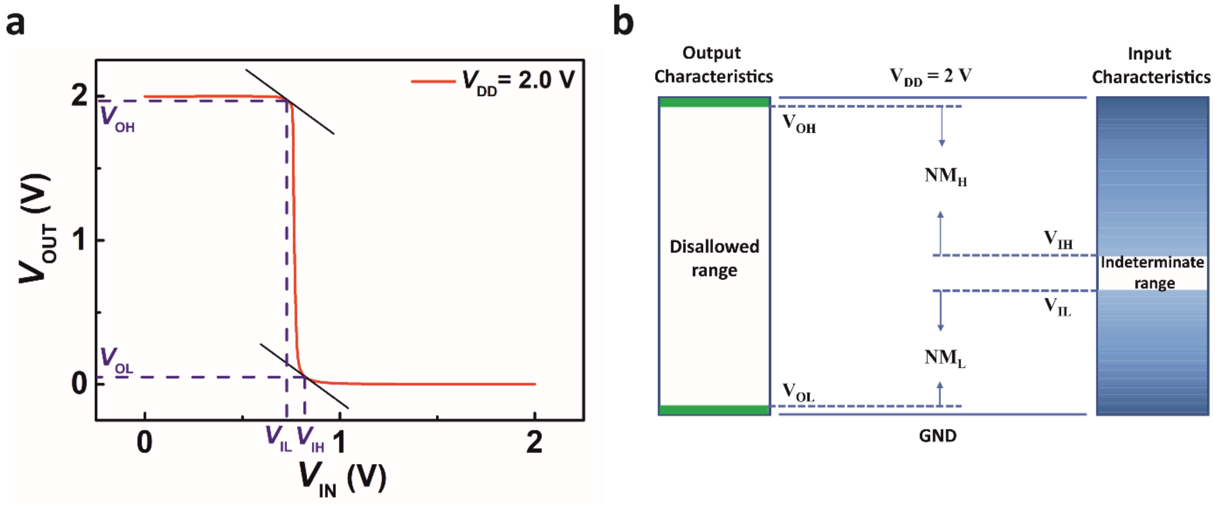

Figure S4: (a) Representative VTC plot for the extraction of V_{OH}, V_{IH}, V_{OL}, V_{IL} which are then used for the calculation noise margin; (b) representative schematic of noise margin of the depletion-load type inverter.

Here, the calculation of noise margin for the depletion-load type inverter has been carried out with the extraction of V_{OH}, V_{IH}, V_{OL}, V_{IL} values (as shown in Figure S4a) from the VTC plot of the inverter, at supply voltage of 2 V, where the slope $\partial V_{OUT}/\partial V_{IN} = -1$. The noise margin value is the calculated as NM_H= V_{OH}-V_{IH} = 1.14 V and NM_L= V_{IL}–V_{OL} = 0.68 V. It is to be noted that a high noise margin value immunes the inverter device from the external noise, which helps to build large circuits consisting of many stages of inverter. From Figure S4a, it is evident that the difference between V_{IH} and V_{IL} is very low, resulting in small the indeterminate voltage window This is necessary for the circuits to avoid the indeterminate states. This is also an essential requirement to build multiple stages for the circuit.

F) The electrophysiological measurement with EG-TFTs based depletion-load type inverters.

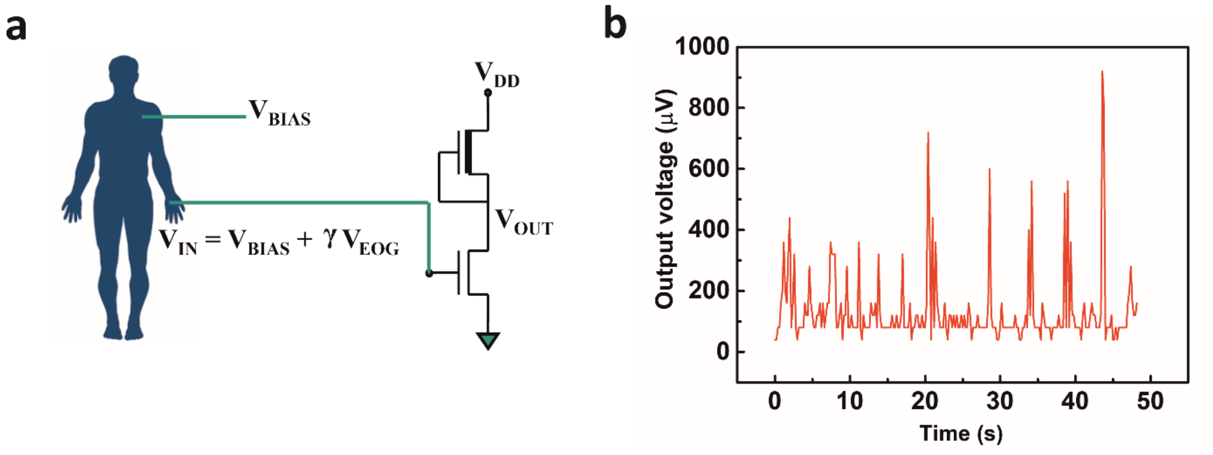

Figure S5: (a) A schematic showing the electrophysiological measurement setup; (b) the output voltage in response to the movement of the hand.

The deep subthreshold operation of the printed *a*-IGZO TFTs enables realization of high gain inverter or high amplification ratio amplifiers, thereby opening possibility of such inverters to be used to detect very weak electrophysiological signals. As shown in Figure S5a, there are two electrodes connected to the body part; while, one electrode is connected to the palm of the hand (lower elbow), the other electrode is connected to the upper elbow position. Here, the upper elbow electrode is connected to the DC bias supply for the amplifier, whereas the lower elbow electrode is connected to the input of the amplifier. Now, when the hand movement occurs, there is an AC signal generated from the body movement coupled with the background DC bias signal is applied to the input of the amplifier, and the amplifier amplifies the body signal, which is in the few μV range to close to mV, as the amplifier output signal shown in Figure S5b. From the above observation, it is evident that these deep-subthreshold operated TFTs and amplifiers are quite befitting to the electrophysiological signal amplification requirements, both in terms of power consumption, as well as the signal amplification ratio required for easy visualization of the weak body signals.

G) A schematic showing the entire smart sensor patch with on-chip power source, sensor, amplifier, ADC, the current drive circuit, and the visual recognition unit.

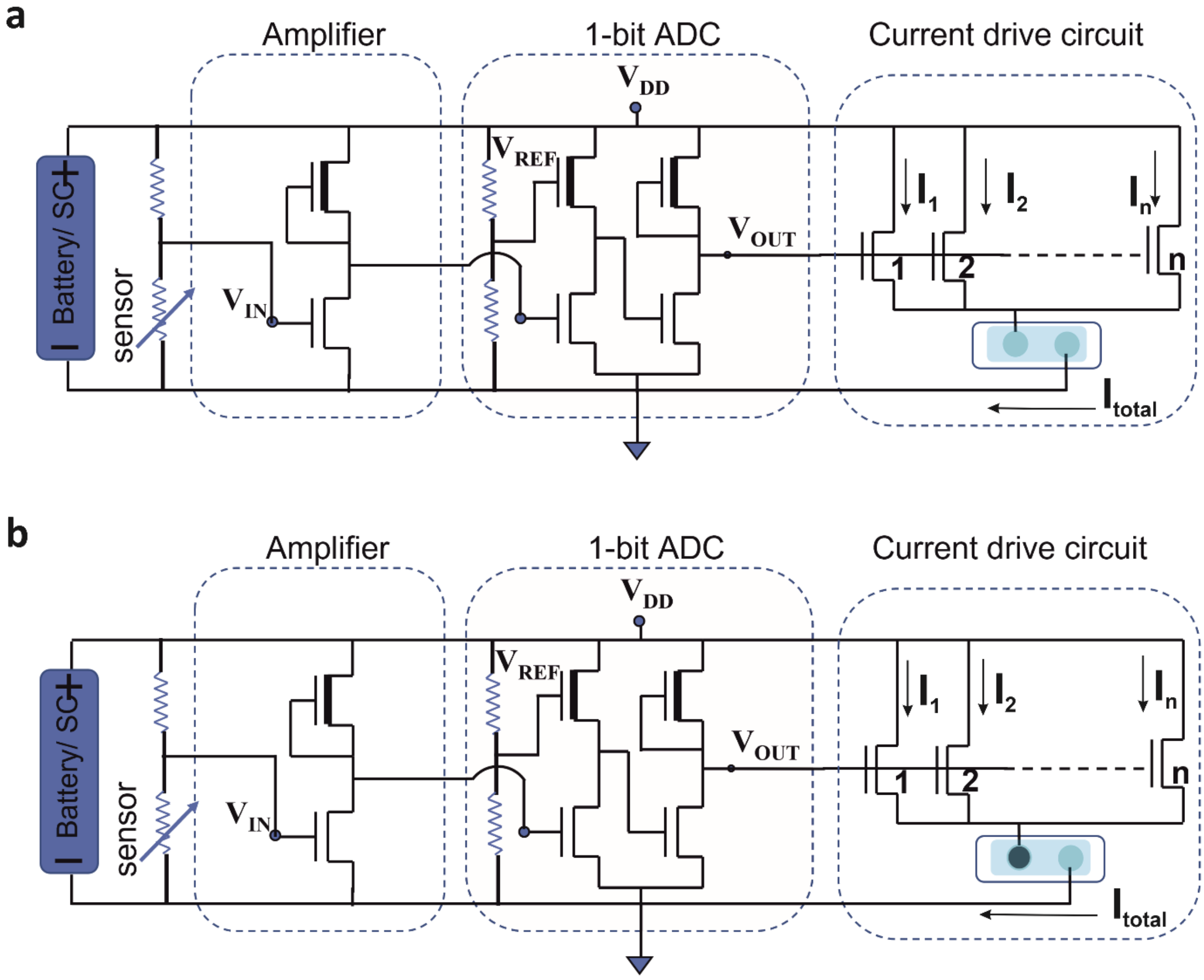

Figure S6: A schematic of a fully printed sensor patch including printed components, such as power source (battery/ supercapacitor), circuits, and visual recognition element. (a) no change in visual recognition element, when the signal from sensor is within the limit decided by V_{REF} and ADC output that is fed to the gate of the current drive circuit TFTs is zero; (b) sudden change in the visual recognition element, when the sensor crosses the limit/threshold decided by V_{REF} causing the digitalized ADC output to go to the high state, this switches the current drive circuit TFTs to on-state causing the entire potential drop alongside a high current to be applied on the visual recognition element.

A schematic of the entire smart sensor patch with on-chip power source is shown in Figure S6. The idea is to interface a large range of sensors, including humidity, temperature, pressure, proximity, chemical, gas, pH, volatile organic compounds (VOCs), biosensors etc. with the amplifier, ADC, the current drive circuit and the visual recognition unit. Any resistive sensor functions as a variable resistor. Therefore, when an external stimulus would cause a change in the resistance of the sensor, the output voltage from the sensor unit, or the voltage divider unit would change. Now, this being the input of the amplifier unit, and the

amplified signal being the input of the 1-bit ADC, the ADC output would depend of the change in the sensor signal. However, the 1-bit ADC unit can be pre-programmed with the help of the V_{REF} to determine at which sensor signal the ADC would switch from the low state, 0 V to the high state, 2 V. This indicates that when the ADC output is at 0 V, this would be the voltage that is applied on the gate of the current drive circuit TFTs, thereby, the TFTs would be at OFF-state and the entire supply voltage would drop across the current drive circuit TFTs. On the other hand, when the pre-defined threshold of the sensor would be reached (controlled by the V_{REF}), the ADC output connected to the gate of the current drive circuit TFTs would switch from low to high state (e.g. 2 V), the current drive circuit TFTs would be fully at the ON-state, allowing high current and the supply voltage to get applied at the visual recognition unit, resulting chemical change induced colour change and a visual recognition of the fact that the sensor signal has crossed a pre-defined threshold.

Here, the readout electronics circuit contains amplifier, ADC and current drive circuit where the current drive circuit consists of multiple individual TFTs whose gate electrodes are commonly connected to the output of the ADC. So, the state of the output of ADC either high state (2 V) or low state (0 V) will control the flow of current through the current drive circuit. Suppose, if the output of ADC is in high state (2 V), then V_{OUT} will switch on all the current drive circuit which will allow the huge current (I_{total}) to flow through the circuit. The total current (I_{total}) would be the sum of each current component associated with the no. of TFTs involved in current drive circuit. Using this large current, multiple current based application like LED, LASER, motor, electrochromic display, chemical reaction, etc. would be possible. The application would not be limited to above mentioned examples; it would be depending upon the user requirements.

(H) The transfer and output characteristics of the EG-TFTs used in the ADC unit, fabricated with 0.03 M *a*-IGZO precursor ink with *W*/*L*= 50 µm/ 50 µm.

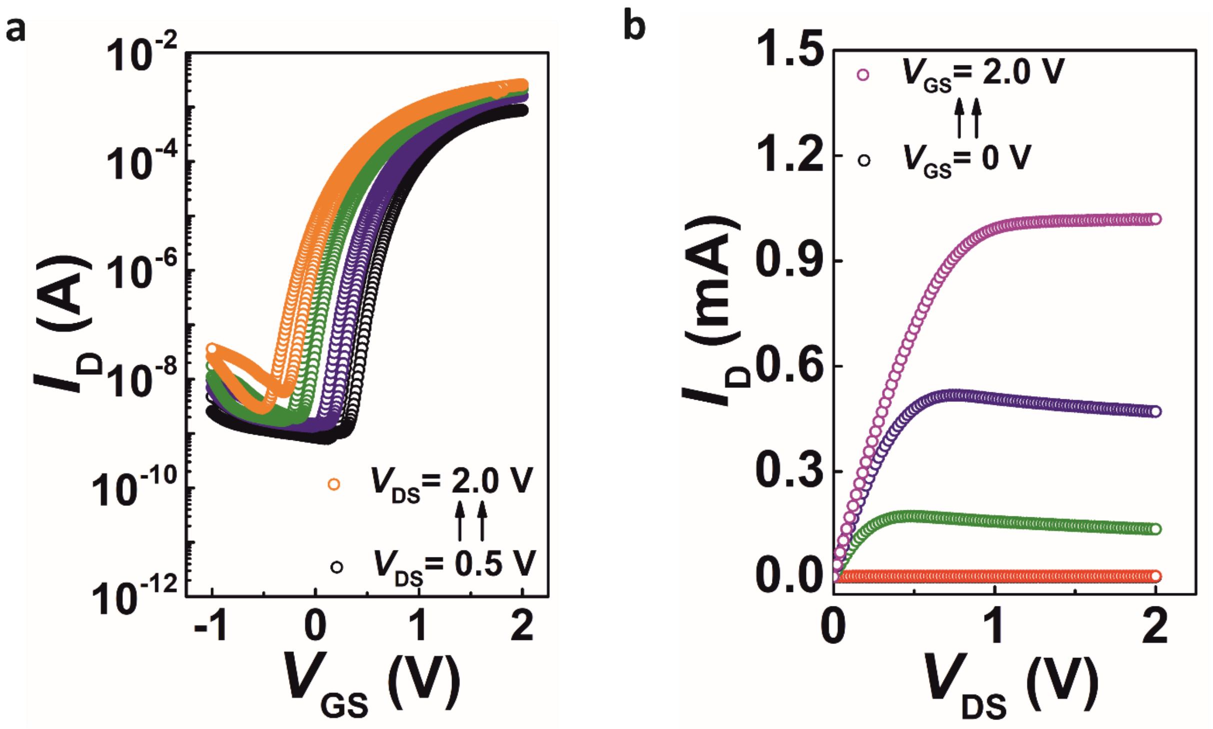

Figure S7: (a) The transfer characteristics, and (b) output characteristics of electrolyte-gated *a*-IGZO based TFT.

Here, the transfer and output characteristics of a representative *a*-IGZO based EG-TFT is shown with device aspect ratio *W*/*L*= 50 µm/ 50 µm, which is being used in ADC circuit, where V_{REF} has been provided for proper functioning of the ADC circuit. It is known that transistors can also act as trans-resistor or variable resistor with response to gate voltage; this idea has been used in the present study to fabricate the compact ADC circuit. Therefore, in the ADC circuit, at first stage T1 and T2 TFTs are connected to common supply voltage and ground; together they function as a voltage divider circuit. For example, when V_{REF} is at 0.1 V, the T2 TFT would provide a constant LOAD for the DRIVE TFT T1, and the supply voltage would be divided among these two TFTs, according to the input voltage applied across the T1 TFT. Consequently, when the input voltage is way below threshold voltage of the TFT T1, the resistance provided by T1 will be more compared to T2. This would lead to the different voltage drop across T1 and T2; this indicates that a substantial potential drop would also take place at T1. Next, this voltage drop across T1 would be fed to the second stage of the ADC unit, which would determine the state of the entire ADC circuit. At first, with V_{REF} at constant 0.1 V, the state of the ADC would be at LOW (0 V), when input voltage at T1 is operated below the threshold voltage. The state of the ADC unit would go to HIGH state (2 V), when the input voltage at T1 is operated above the threshold voltage. This transition point of ADC state would vary depending upon the variation in the reference voltage value. In this case, due to the fact that the reference voltage is applied at T2, T2 TFTs acts as a variable LOAD for the DRIVE TFT T1.

I) The voltage transfer characteristics (VTC) of the *a*-IGZO EG-TFT based analog-to-digital converter (ADC), re-measured after 1 month.

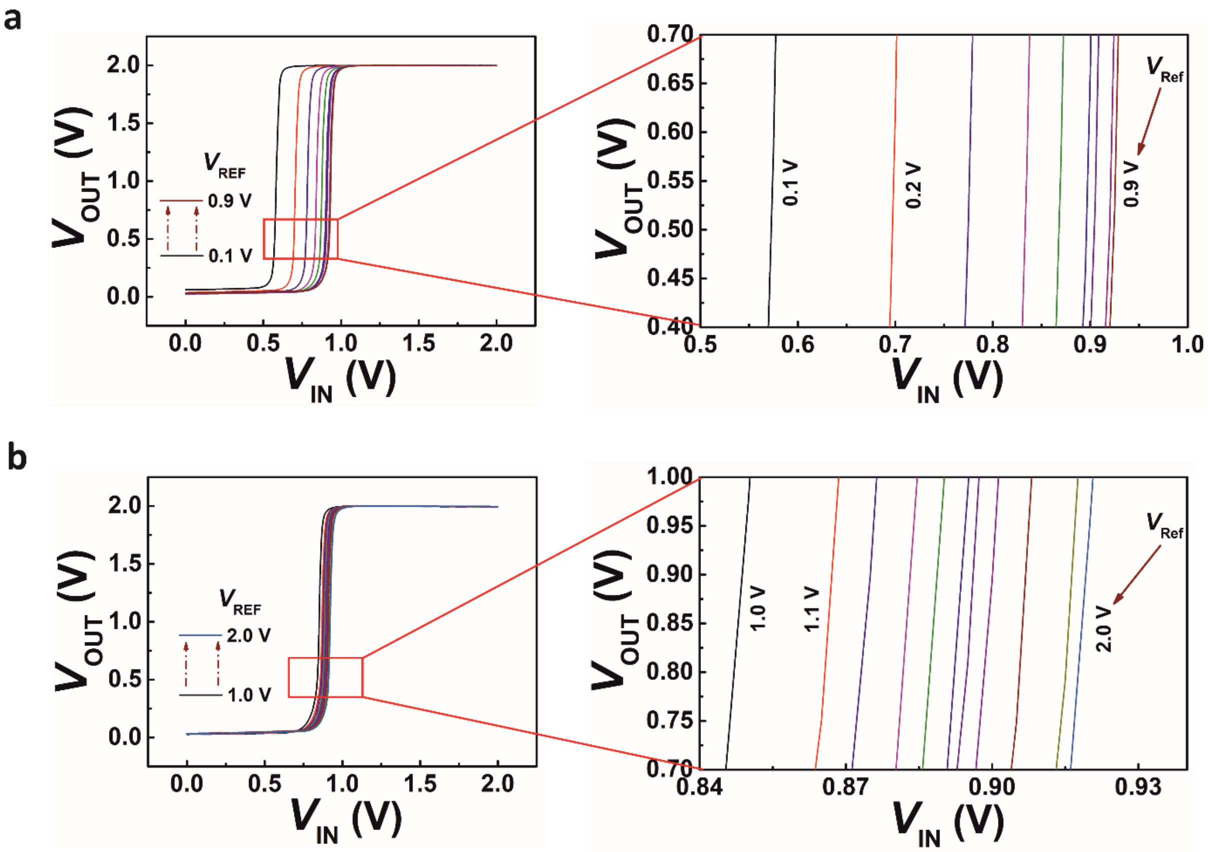

Figure S8: The VTC curve of ADC with a varying reference voltage V_{REF} from (a) 0.1 V to 0.9 V; (b) 1.0 V to 2.0 V, with a voltage step size of 0.1 V.

J) The VTC curve of the ADC alongside an amplifier, re-measured after 1 month.

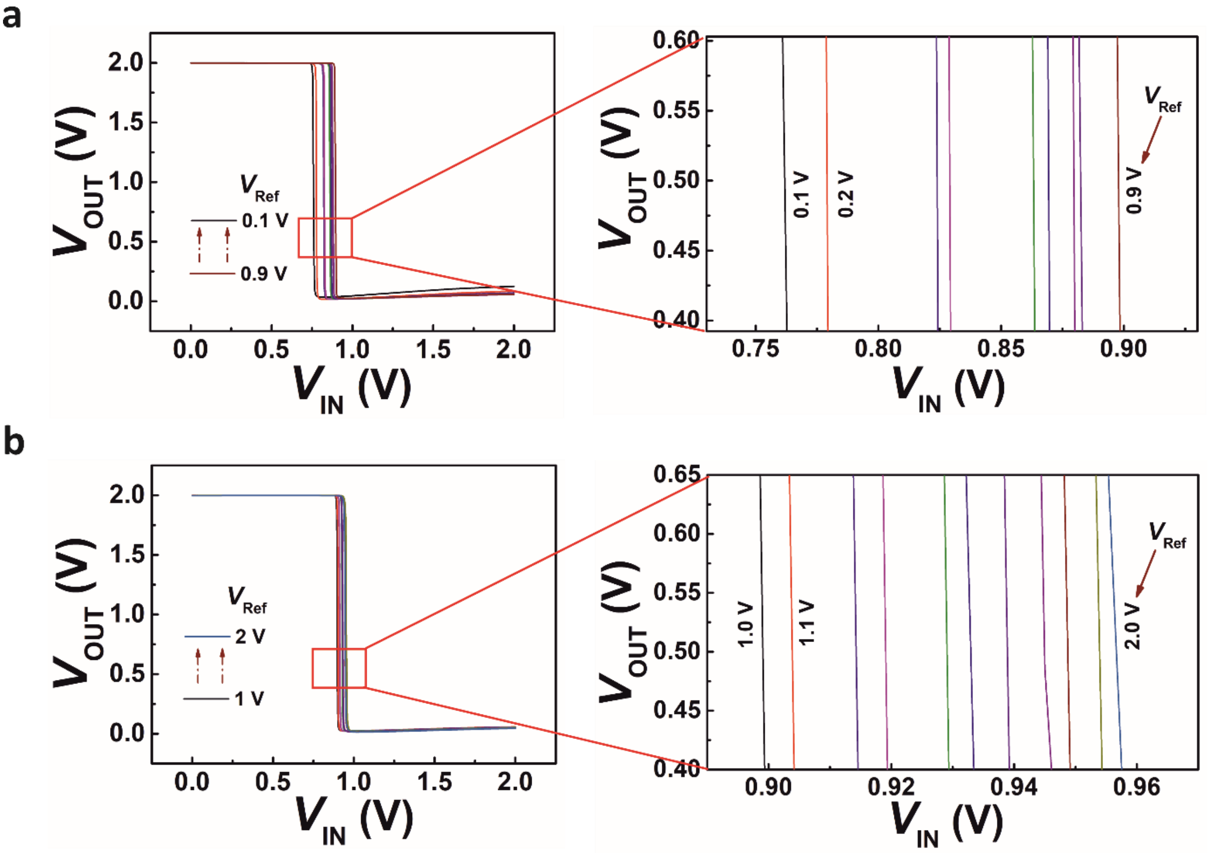

Figure S9: The VTC curve of amplifier and ADC circuit, with varying the reference voltage V_{REF} from (a) 0.1 V to 0.9 V; (b) 1.0 V to 2.0 V with a voltage step size of 0.1 V.

K) The dynamic response of the ADC alongside the amplifier with a triangular analog input.

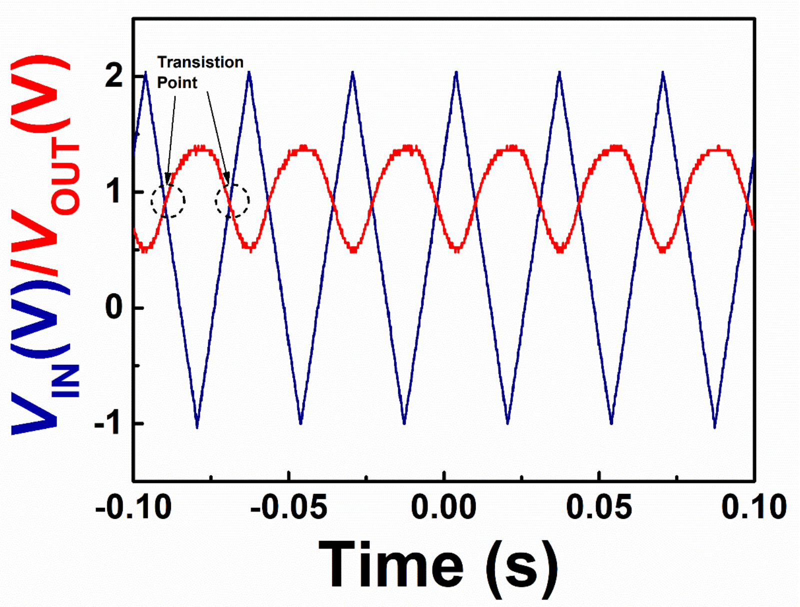

Figure S10: Dynamic response of the ADC unit alongside the common-source amplifier, recorded at 30 Hz.

When the ADC unit along with the common-source amplifier is provided with the triangular waveform at the input, the output of the ADC switches its state according to the particular input voltage at a fixed reference voltage V_{REF}= 1.0 V.

L) The gate current of a typical *a*-IGZO based EG-TFT device.

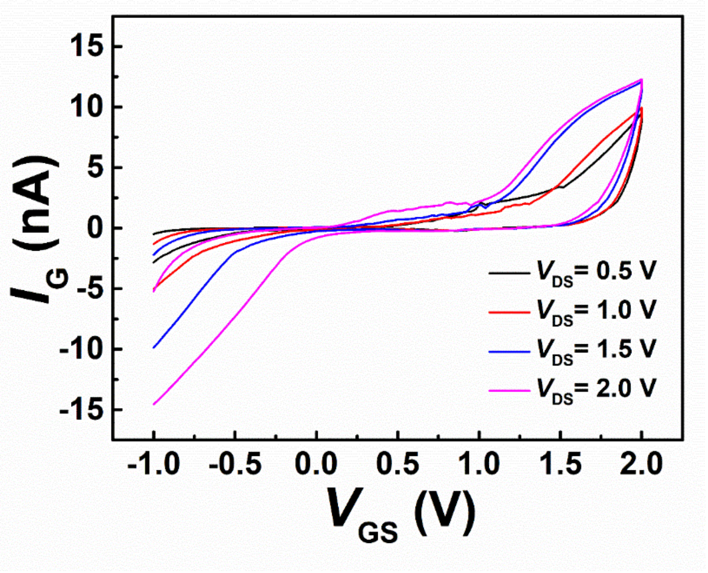

Figure S11: Gate current (I_G) vs. gate voltage of a typical a-IGZO based EG-TFT device.

The gate current (I_G) is recorded for a typical *a*-IGZO based EG-TFT device. As the gate current of the EG-TFTs is in the range of 10-15 nA, the value of the resistance of the fixed resistor or the variable resistor (i.e. the sensor) may easily be up to MOhm range. This means that when the sensor unit or the voltage divider units total resistance will be up to MOhm range, the flow of majority of the current will be through it, thereby catching actual voltage variation across the sensor, not through the gate of the drive TFT of the amplifier unit, where the input voltage from the sensor unit has been fed in.

Next, a voltage divider circuit consisting of a series connection of a fixed resistor of 10 kOhm and variable resistor is connected across the common supply voltage (V_{DD}), and the common ground. Here, the intermediate voltage of the voltage divider circuit, which is in fact the potential drop across the variable resistor, gets fed into the ADC, with the amplifier unit in between. That voltage drop across the variable resistor determines the state of the output voltage at a particular reference voltage (V_{REF}). From the VTC of the entire circuit, it is clear that with changing the V_{REF}, the transition point for the output voltage from HIGH to LOW can be shifted. Therefore, when we change the V_{REF} value in the circuit, indirectly the potential (drop across variable resistor) required at the input for changing the state of the output voltage will change. That can be achieved via changing the resistance value of the variable resistor. It means that for a particular V_{REF} value, there is a fixed resistance value assigned at the input. This is called discretisation of the input voltage, which is a function of the designed ADC circuit. With similar analogy, instead of a variable resistor, an actual resistive sensor, such as humidity, gas, proximity, temperature, chemical, etc., which act as a variable resistor with respect to the response to an external stimulus can be connected to this circuit for the discretisation of the input potential value that would be fed to the amplifier and ADC unit.

Table S3: The value of variable resistance in response to the reference voltage.

V_{REF} (V)	Variable Resistance (kOhm)
0.4	2.1
0.6	3.3
0.8	3.8
1.0	3.9

M) The printed ADC with amplifier circuit interfacing with the real world: connected to ARDUINO and ESP32 wireless board.

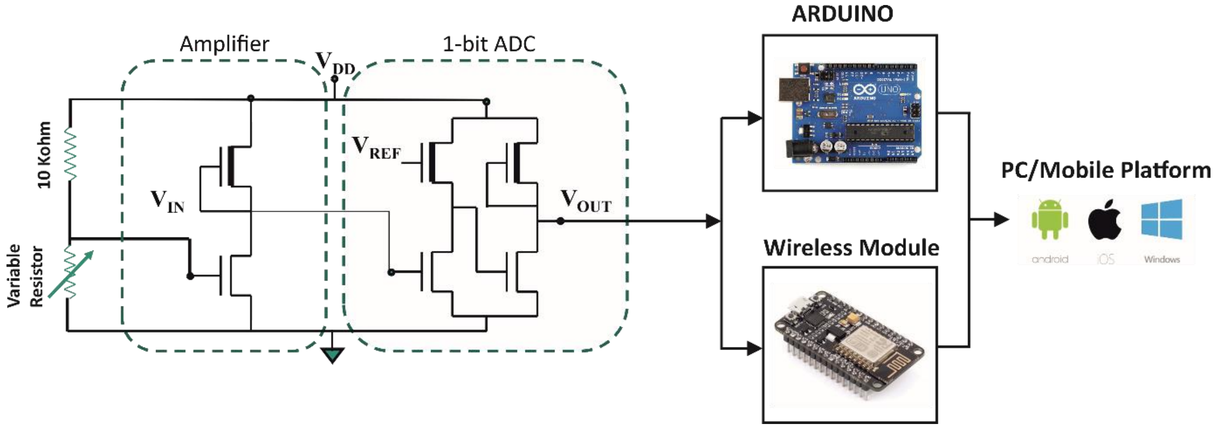

Figure S12: A schematic of the representation of the hybrid electronics, where the ADC and the amplifier are printed circuits and are connected to ARDUINO and ESP32 wireless board.

In order to study the compatibility of the printed readout electronics with standard electronic components, the readout electronic circuit has been connected to the ARDUINO board, where it also performs in an identical manner. One of the major concerns about the printed electronic devices and sensors is that most of these devices require further amplification of the obtained signals in order to make the sensor signal understandable. In fact, the further amplification of these weak signals from the printed devices requires additional circuit, which makes the sensing system more complex. The introduction of discrete/ lumped silicon-based devices for amplification on same chip, on which the sensors are printed, is quite cumbersome, when the used substrate is glass or PET. Furthermore, these comparatively bulky silicon chips may result in the flexible substrates to lose their application edge, by making them partially rigid and heavy. On the other hand, another problem with the bulky Si chips on the identical flexible tag is that an elevated temperature would be required for soldering, which may be higher than most of the inexpensive flexible substrates can withstand.

This problem can be solved by using printed circuits that bridge the gap between the printed sensors and the real-world, by providing sufficient strong signals in terms of voltage and currents, which can be directly used for human interaction and understanding. Here, the printed circuit, which consists of an amplifier and a 1-bit analog-to-digital converter (ADC) may potentially be used with any printed sensor system to make the data readable by a standard microcontroller, which may further be processed for user interface without requiring any additional amplification stage. In this printed circuit, the data obtained from a variable resistor, which is analogous to a resistive sensor, is amplified by a depletion-load type unipolar inverter-based common-source amplifier, and further converted into a digital signal using an ADC circuit. The output obtained from the printed 1e-bit ADC can be directly fed into these microcontrollers' pin and further processed to either indicate user by turning LEDs On and Off, or by transmitting it wirelessly to a mobile phone or PC, as shown in Figure S12.

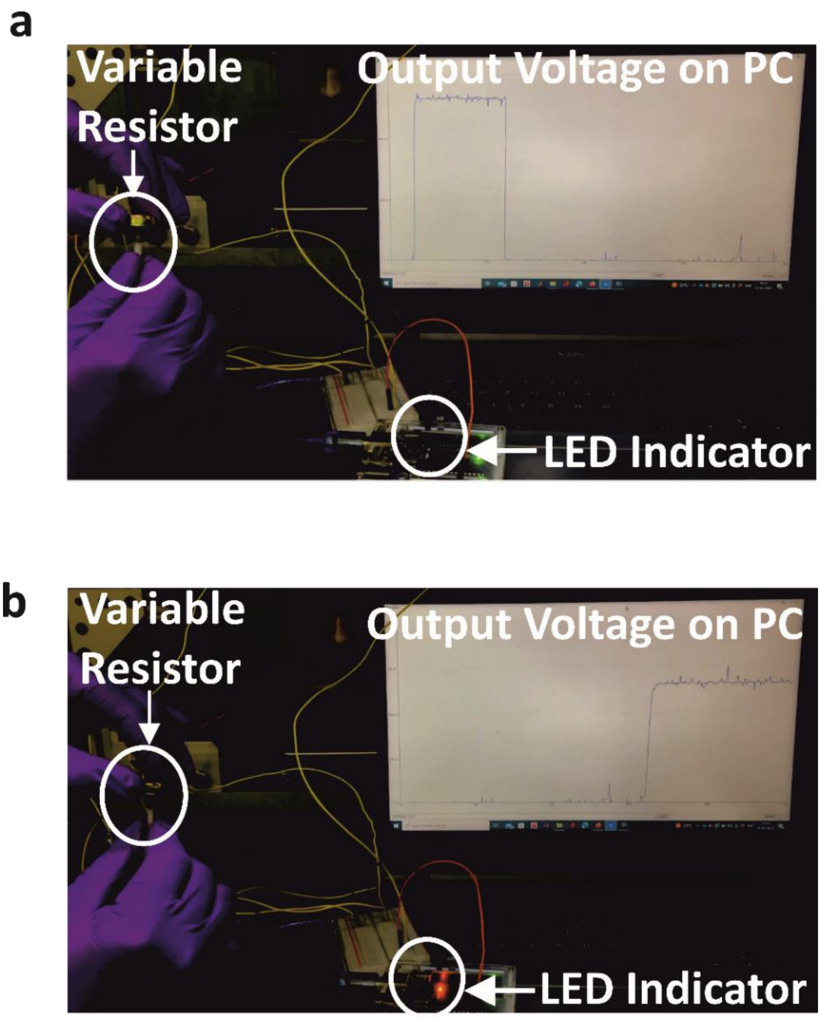

Figure S13: (a) The output voltage from the ADC with an amplifier circuit becomes zero in response to the change in the resistance value of the variable resistor is recorded on a PC via an ARDUINO board, and have also been indicated by the OFF-state of the LED attached to it; (b) the output voltage from the ADC with an amplifier circuit turns to the HIGH state (2 V) in response to the change in the resistance value of the variable resistor is being recorded on a PC via the ARDUINO board, and have also been indicated by the ON-state of the LED on the ARDUINO board.

Here, the compatibility of the above-shown printed circuit output with two different microcontrollers is demonstrated. At first, we use an Arduino UNO board to turn On-and-Off the inbuilt LED on the board based on the signal obtained from the printed circuit, while changing the resistance of the variable resistor continuously, resulting in a change in the applied voltage across the input of the printed amplifier and ADC circuit, which then decides the output of the circuit to be either at the HIGH-state (2 V- LED- ON) or at the LOW-state (0 V- LED- OFF), as shown in Figure S13. In another instance, we use an ESP32 board to transmit the ADC data wirelessly to an android app in a mobile phone, shown in Figure S14. The measured values of the ADC unit using each of the boards were found to be equal to what is measured by the oscilloscope. This suggests that the ADC circuit output impedance does not result in any mismatch or loading effects, when connected to various external circuits.

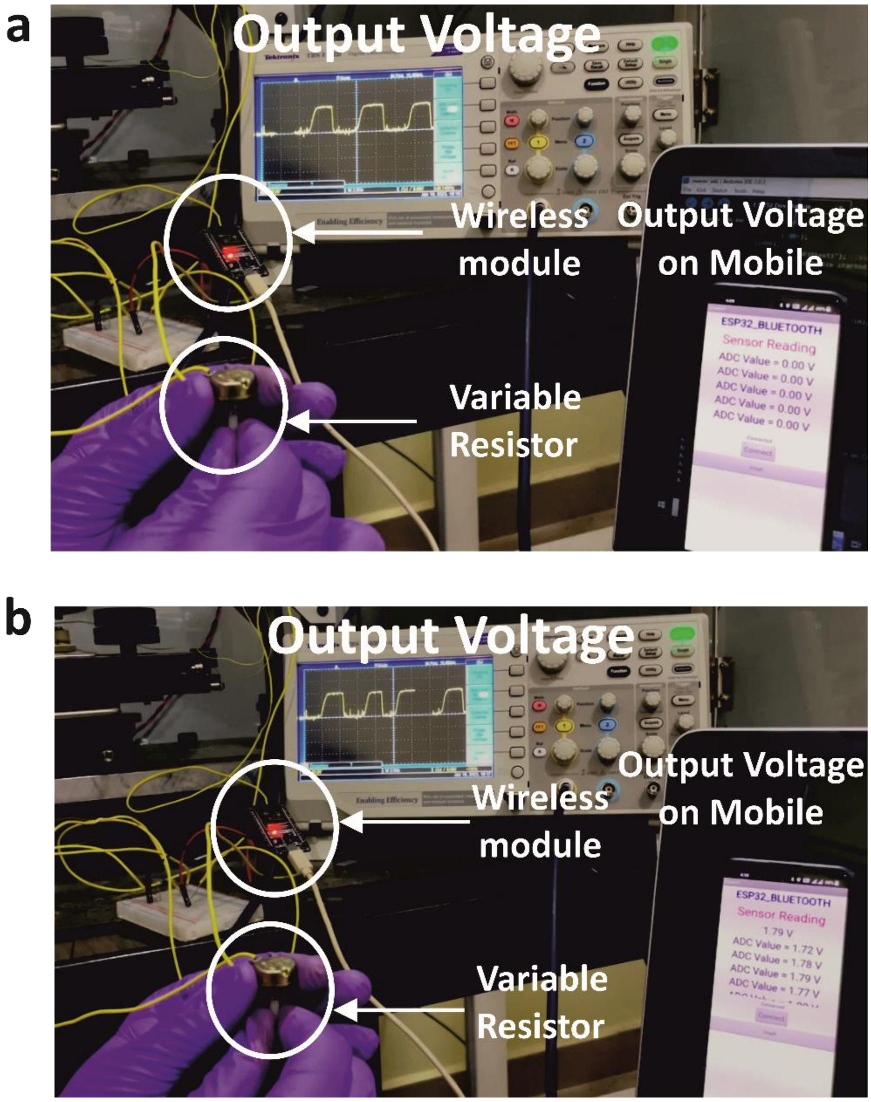

Figure S14: (a) The output voltage from the ADC unit, with an amplifier circuit turns LOW (zero) in response to a resistance variation in the variable resistor is being observed on an android mobile, via an ESP32 wireless module; (b) the output voltage from the ADC with an amplifier circuit turns HIGH (nearly 2 V) in response to a resistance variation in the variable resistor is being observed on an android mobile phone, via an ESP32 wireless module.

N) Synthesis of Mxene and Mxene-based temperature sensor.

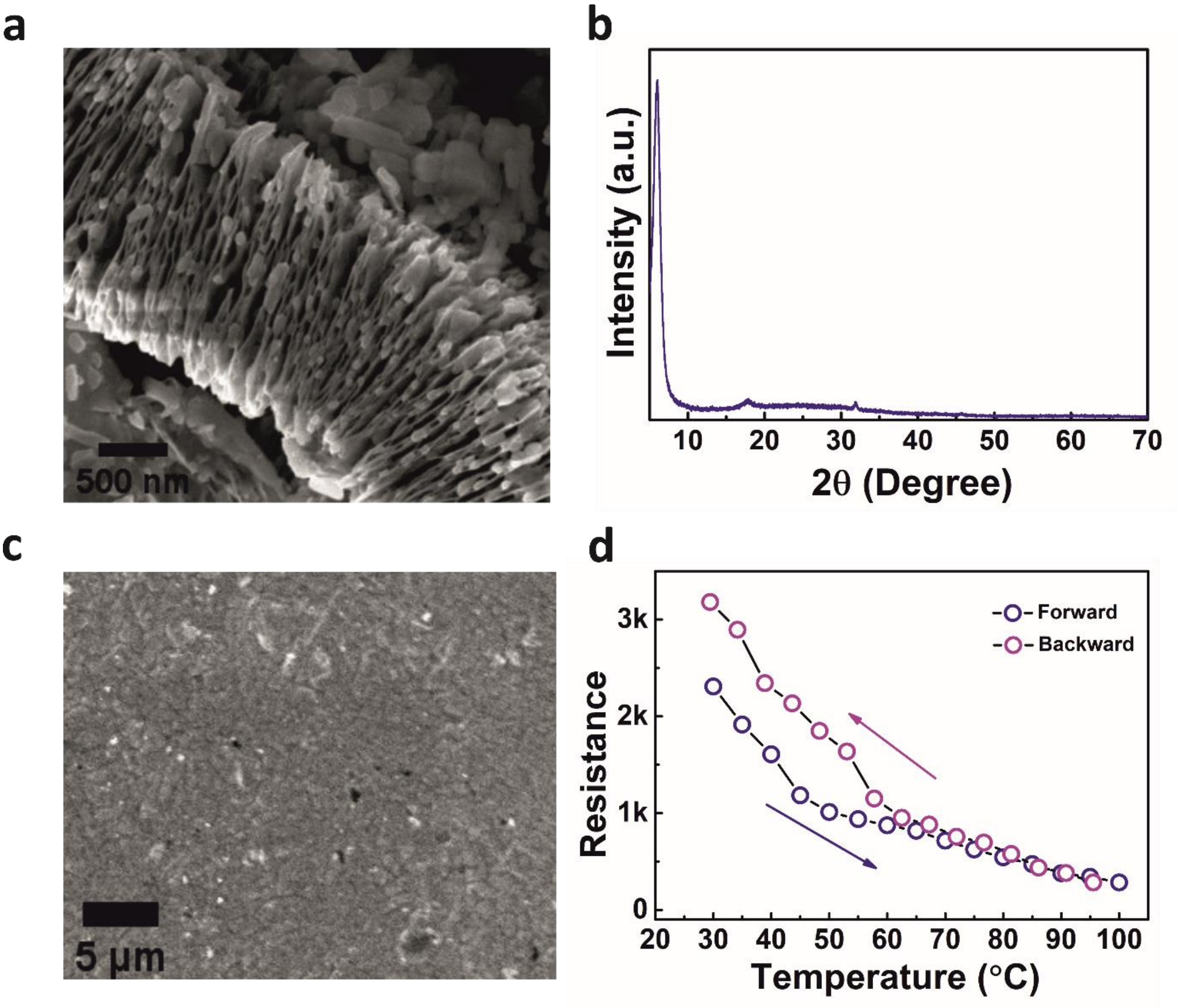

Figure S15: (a) SEM micrograph showing etched MXene flakes; (b) XRD pattern of a synthesized MXene; (c) SEM micrograph of the mixture of MXene, PVA and PVP. (d) temperature sensor performance of the MXene-based sensor, resistance change in the forward and backward temperature sweep.

***Synthesis of Mxene*:**

In order to synthesize $Ti_3C_2T_x$, required amount of 0.8 g LiF was added 10 ml of 9 M HCl and kept stirring for about 5 minutes. After complete mixing, 0.5 g of MAX powder (Ti_3AlC_2) was added to the solution and stirred for about 24 h at 40 °C. To get rid of the by-products of the reactions, the mixture was then washed with copious amount of DI water for several times until the pH was normalized. Next, the mixture was bath sonicated for 1 h and centrifuged at 6500 rpm for 5 minutes, and the supernatant was collected as highly exfoliated 2d $Ti_3C_2T_x$. Single phase as-etched $Ti_3C_2T_x$ flakes with accordion-like microstructure has been observed from Figure S15a and Figure S15b.

***Fabrication of Temperature sensor*:**

To fabricate the temperature sensor based on Mxene, 30 wt.% PVA was dissolved in DI water at 100 °C, and 20 wt.% polyvinyl pyrolidene (PVP) was dissolved in DI water

separately. Next, both the solutions were mixed and stirred for 2 h. Subsequently, exfoliated Mxene was added to the solution to achieve a Mxene loading of 3 mg/ml. To polymerise the solution, 1 ml H_2SO_4 was added and then the whole solution/slurry was transferred to a petri dish and kept it at -20 °C for 12 h for refrigeration and then defrosted by bringing it to room temperature for 6 h. The refrigeration and de-freezing cycle was repeated for 3 times in order to achieve a gel form. Figure S15c shows the SEM micrograph of temperature sensor made up of PVA, PVP and MXene. It is clear from SEM micrograph that the MXene blended with the polymer mixture of PVA and PVP completely. After fabricating the temperature sensor, it has undergone the resistance measurement with respect to temperature. It is observed that with increase in temperature, the resistance decreases that shows negative temperature coefficient behaviour of the temperature sensor (shown in figureS15d).

O) Temperature sensor measurement with printed circuit set at critical temperature of 32 °C.

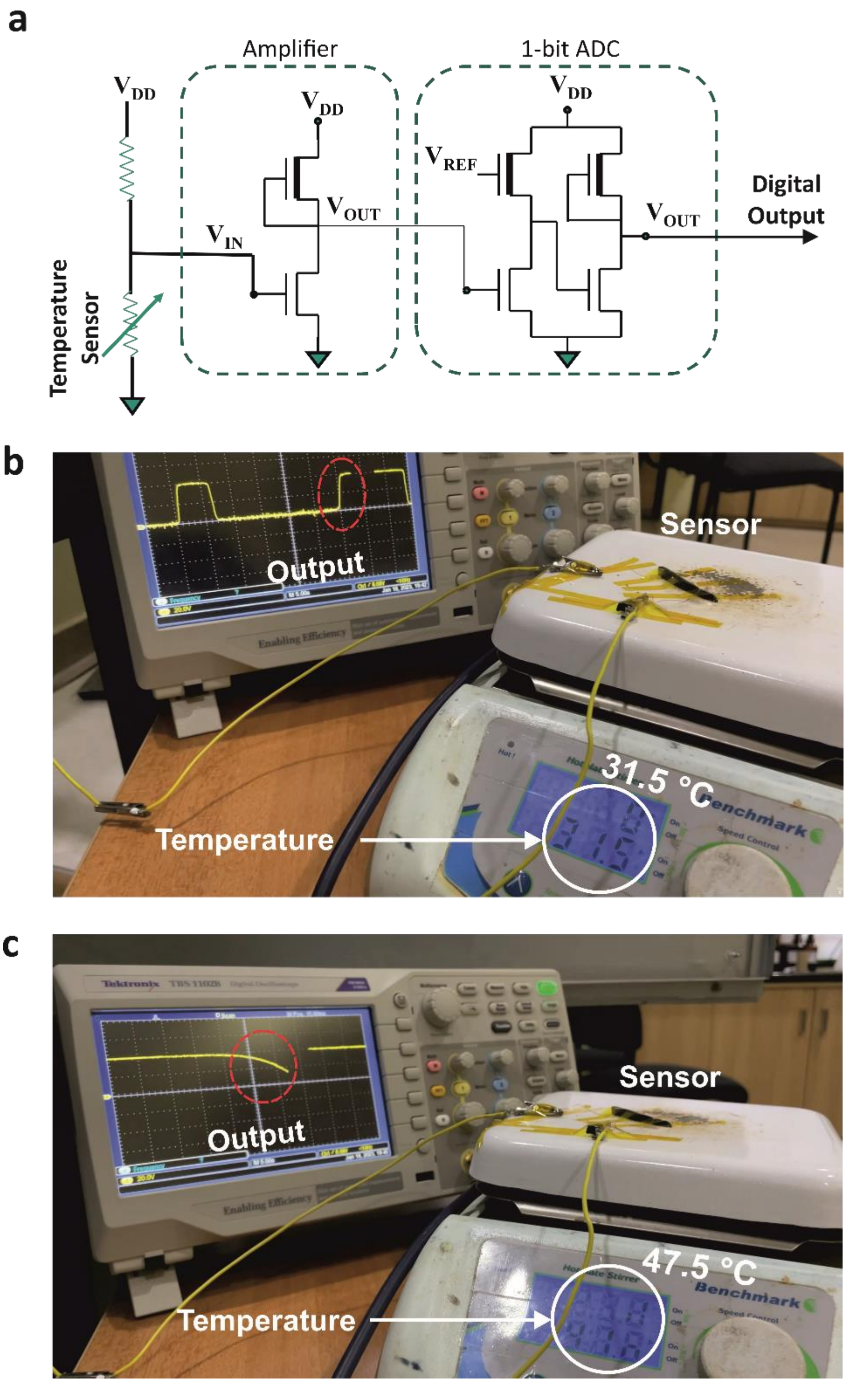

Figure S16: (a) Setup of temperature sensor connected to ADC with an amplifier circuit (b) the output voltage becomes high when the temperature crosses 32 °C (indicated by red dot circle) (c) the output voltage becomes low when the temperature decreases below 47 °C (indicated by red dot circle).

P) A schematic of a multi-bit ADC circuit.

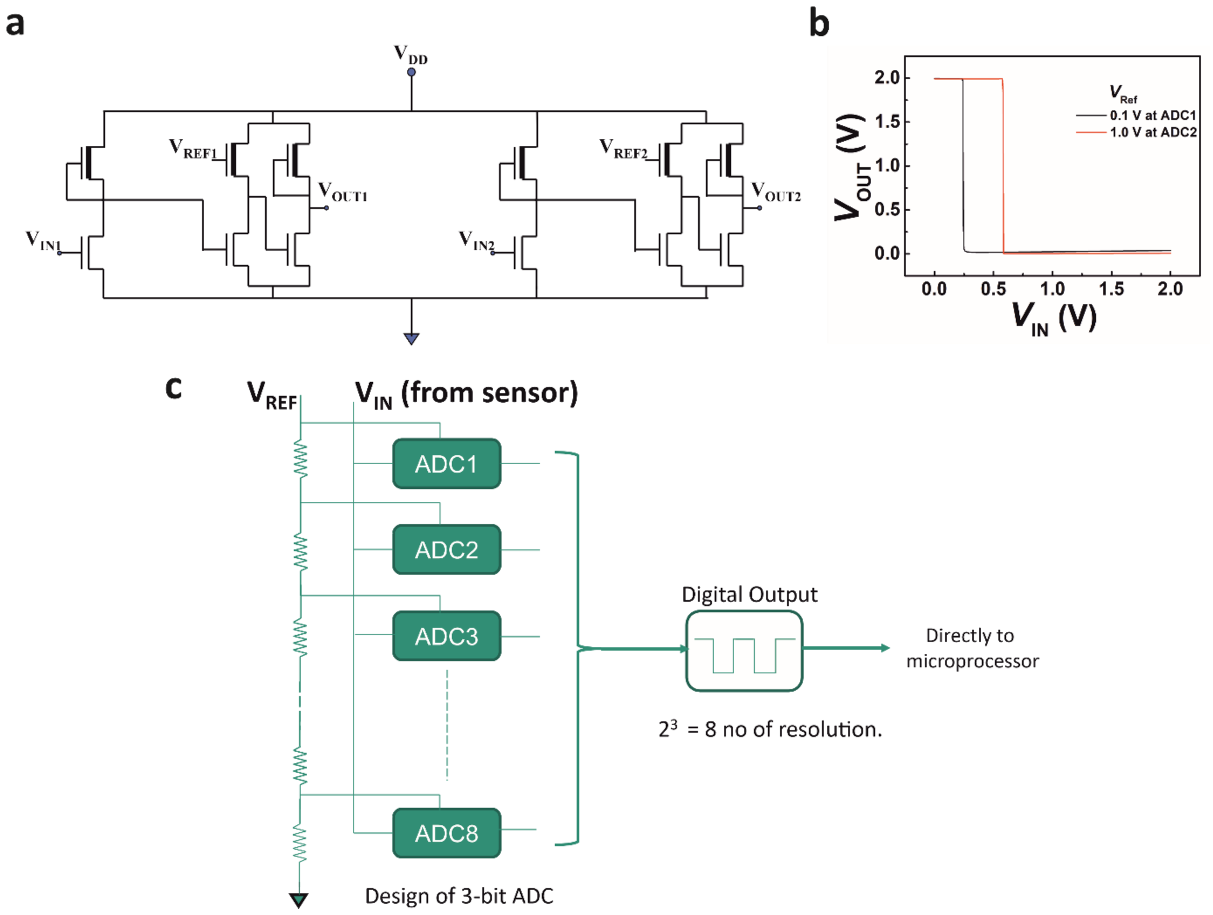

Figure S17: (a) A schematic of two ADC circuits that are connected in parallel and supplied with identical supply voltage; (b) The VTC curve of the circuit where two reference voltages are applied to two different ADC units (c) A schematic of multibit ADC circuit.

The Figure S17 shows the schematic of multi-bit operation using proposed ADC circuit. When the individual ADCs are connected in parallel with common supply voltage and ground potential, it can operate as multi-bit ADC unit. For example, 3-bit ADC circuit connection requires $2^n = 2^3 = 8$ ADCs and $2^n - 1 = 2^3 - 1 = 7$ resistors.

Q) UV-visible spectroscopy of the device.

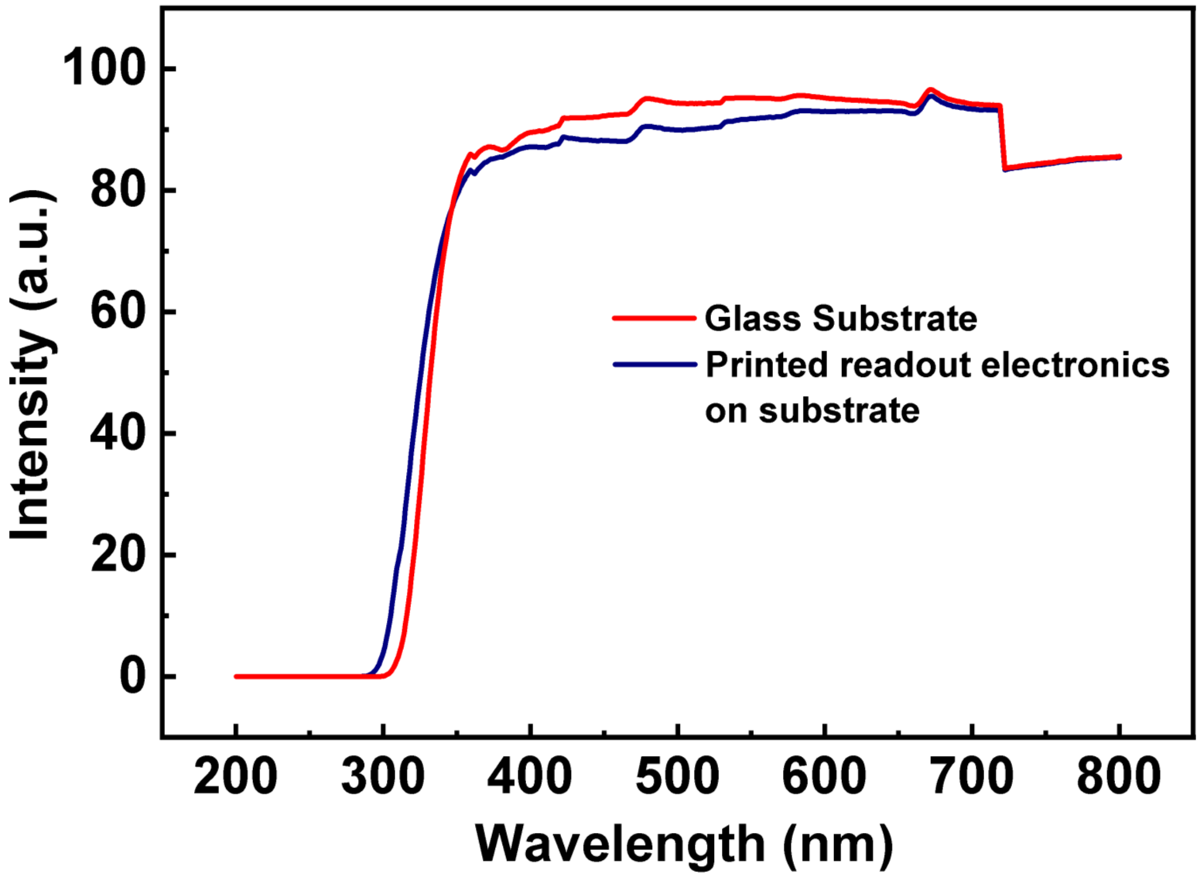

Figure S18: UV-visible spectroscopy of the printed circuit on glass substrate.

The collected UV-Visible spectroscopy data show that printed readout electronics that include the transparent conducting oxide ITO, the transparent oxide semiconductor *a*-IGZO, the transparent electrolytic insulator CSPE and the organic metal PEDOT:PSS is together nearly as transparent as the bare glass substrate used for the device fabrication. Therefore, the printed smart sensor tag may also classify as smart transparent electronics.
